# A Convex Variational Model for Learning Convolutional Image Atoms from Incomplete Data

**DOI:** 10.1007/s10851-019-00919-7

**Published:** 2019-11-18

**Authors:** A. Chambolle, M. Holler, T. Pock

**Affiliations:** 1 Centre de Mathématiques Appliquées, École Polytechnique, Paris, France; 2 Institute of Mathematics and Scientific Computing, University of Graz, Graz, Austria; 3 Institute of Computer Graphics and Vision, Graz University of Technology, Graz, Austria

**Keywords:** Variational methods, Learning approaches, Inverse problems, Functional lifting, Convex relaxation, Convolutional Lasso, Machine learning, Texture reconstruction, 94A08, 49M29, 65F22, 49K30

## Abstract

A variational model for learning convolutional image atoms from corrupted and/or incomplete data is introduced and analyzed both in function space and numerically. Building on lifting and relaxation strategies, the proposed approach is convex and allows for simultaneous image reconstruction and atom learning in a general, inverse problems context. Further, motivated by an improved numerical performance, also a semi-convex variant is included in the analysis and the experiments of the paper. For both settings, fundamental analytical properties allowing in particular to ensure well-posedness and stability results for inverse problems are proven in a continuous setting. Exploiting convexity, globally optimal solutions are further computed numerically for applications with incomplete, noisy and blurry data and numerical results are shown.

## Introduction

An important task in image processing is to achieve an appropriate regularization or smoothing of images or image-related data. In particular, this is indispensable for most application-driven problems in the field, such as denoising, inpainting, reconstruction, segmentation, registration or classification. Also beyond imaging, for general problem settings in the field of inverse problems, an appropriate regularization of unknowns plays a central role as it allows for a stable inversion procedure.

Variational methods and partial-differential-equation-based methods can now be regarded as classical regularization approaches of mathematical image processing (see, for instance, [5, 42, 54, 62]). An advantage of such methods is the existence of a well-established mathematical theory and, in particular for variational methods, a direct applicability to general inverse problems with provable stability and recovery guarantees [31, 32]. While in particular piecewise smooth images are typically well described by such methods, their performance for oscillatory- or texture-like structures, however, is often limited to predescribed patterns (see, for instance, [27, 33]).

Data-adaptive methods such as patch- or dictionary-based methods (see, for instance, [2, 13, 22, 23, 37]) on the other hand are able to exploit redundant structures in images independent of an a priori description and are, at least for some specific tasks, often superior to variational- and PDE-based methods. In particular, methods based on (deep) convolutional neural networks are inherently data adaptive (though data adaptation takes place in a preprocessing/learning step) and have advanced the state of the art significantly in many typical imaging applications in the past years [38].

Still, for data-adaptive approaches, neither a direct applicability to general inverse problems nor a corresponding mathematical understanding of stability results or recovery guarantees are available to the extend they are with variational methods. One reason for this lies in the fact that, for both classical patch- or dictionary-based methods and neural-network-based approaches, data adaptiveness (either online or in a training step) is inherently connected to the minimization of a non-convex energy. Consequently, standard numerical approaches such as alternating minimization or (stochastic) gradient descent can, at best, only be guaranteed to deliver stationary points of the energy, hence suffering from the risk of delivering suboptimal solutions.

The aim of this work is to provide a step toward bridging the gap between data-adaptive methods and variational approaches. As a motivation, consider a convolutional Lasso problem [39, 65] of the form

1 $$\begin{aligned} \min _{(c_i)_i,(p_i)_i} \lambda \mathcal {D} \left( \sum _{i=1}^k c_i * p_i, u_0\right) + \sum _{i=1}^k \Vert c_i\Vert _1 \quad \text {s.t. } p_i \in \mathcal {C} .\nonumber \\ \end{aligned}$$

Here, the goal is to learn image atoms $(p_i)_i$ (constrained to a set $\mathcal {C}$) and sparse coefficient images $(c_i)_i$ which, via a convolution, synthesize an image corresponding to the given data $u_0$ (with data fidelity being measured by $\mathcal {D}$). This task is strongly related to convolutional neural networks in many ways, see [46, 61, 65] and the paragraph *Connections to deep neural networks* below for details. A classical interpretation of this energy minimization is that it allows for a sparse (approximate) representation of (possible noisy) image data, but we will see that this synopsis can be extended to include a forward model for inverse problems and a second image component of different structures. In any case, the difficulty here is non-convexity of the energy in (1), which complicates both analysis and its numerical solution. In order to overcome this, we build on a tensorial-lifting approach and subsequent convex relaxation. Doing so and starting from (1), we introduce a convex variational method for learning image atoms from noisy and/or incomplete data in an inverse problems context. We further extend this model by a semi-convex variant that improves the performance in some applications. For both settings, we are able to prove well-posedness results in function space and, for the convex version, to compute globally optimal solutions numerically. In particular, classical stability and convergence results for inverse problems such as the ones of [31, 32] are applicable to our model, providing a stable recovery of both learned atoms and images from given, incomplete data.

Our approach allows for a joint learning of image atoms and image reconstruction in a single step. Nevertheless, it can also be regarded purely as an approach for learning image atoms from potentially incomplete data in a training step, after which the learned atoms can be further incorporated in a second step, e.g., for reconstruction or classification. It should also be noted that, while we show some examples where our approach achieves a good practical performance for image reconstruction compared to the existing methods, the main purpose of this paper is to provide a mathematical understanding rather than an algorithm that achieves the best performance in practice.

*Related Works* Regarding the existing literature in the context of data-adaptive variational learning approaches in imaging, we note that there are many recent approaches that aim to infer either parameter or filters for variational methods from given training data, see, e.g., [14, 30, 36]. A continuation of such techniques more toward the architecture of neural networks is so-called variational networks are so-called [1, 35] where not only model parameters but also components of the solution algorithm such as stepsizes or proximal mappings are learned. We also refer to [41] for a recent work on combining variational methods and neural networks. While for some of those methods also a function space theory is available, the learning step is still non-convex and the above approaches can in general only be expected to provide locally optimal solutions.

In contrast to that, in a discrete setting, there are many recent directions of research which aim to overcome suboptimality in non-convex problems related to learning. In the context of structured matrix factorization (which can be interpreted as the underlying problem of dictionary learning/sparse coding in a discrete setting), the authors of [29] consider a general setting of which dictionary learning can be considered as a special case. Exploiting the existence of a convex energy which acts as lower bound, they provide conditions under which local optima of the convex energy are globally optimal, thereby reducing the task of globally minimizing a non-convex energy to finding local optima with certain properties. In a similar scope, a series of works in the context of dictionary learning (see [4, 55, 59] and the references therein) provide conditions (e.g., assuming incoherence) under which minimization algorithms (e.g., alternating between dictionary and coefficient updates) can be guaranteed to converge to a globally optimal dictionary with high probability. Regarding these works, it is important to note that, as discussed in Sect. 2.1 (see also [26]), the problem of dictionary learning is similar but yet rather different to the problem of learning convolutional image atoms as in (1) in the sense that the latter is shift-invariant since it employs a convolution to synthesize the image data (rather than comparing with a patch matrix). While results on structured matrix decomposition that allow for general data terms (such as [29]) can be applied also to convolutional sparse coding by including the convolution in the data term, this is not immediate for dictionary learning approaches.

Although having a different motivation, the learning of convolutional image atoms is also related to blind deconvolution, where one aims to simultaneously recover a blurring kernel and a signal from blurry, possibly noisy measurements. While there is a large literature on this topic (see [15, 20] for a review), in particular lifting approaches that aim at a convex relaxation of underlying bilinear problem in a discrete setting are related to our work. In this context, the goal is often to obtain recovery guarantees under some assumptions on the data. We refer to [40] for a recent overview, to [3] for a lifting approach that poses structural assumptions on both the signal and the blurring kernel and uses a nuclear-norm-based convex relaxation, and to [21] for a more generally applicable approach that employs the concept of atomic norms [19]. Moreover, the work [40] studies the joint blind deconvolution and demixing problem, which has the same objective as (1) of decomposing a signal into a sum of convolutions but is motivated in [40] from multiuser communication. There, the authors again pose some structural assumptions on the underlying signal but, in contrast to previous works, deal with recovery guarantees of non-convex algorithms, which are computationally more efficient than those addressing a convex relaxation.

*Connections to Deep Neural Networks* Regarding a deeper mathematical understanding of deep convolutional neural networks, establishing a mathematical theory for convolutional sparse coding is particularly relevant due to a strong connection of the two methodologies. Indeed, it is easy to see that for instance in case $$ \mathcal {D} (u,u0) = \frac{1}{2}\Vert u-u_0\Vert _2 ^2$$ and $$(p_i)_i $$ is fixed, the numerical solution of the convolutional sparse coding problem (1) via forward-backward splitting with a fixed number of iterations is equivalent to a deep residual network with constant parameters. Similarly, recent results (see [46, 47, 58]) show a strong connection of thresholding algorithms for multilayer convolutional sparse coding with feed-forward convolutional neural networks. In particular, this connection is exploited to transfer reconstruction guarantees from sparse coding to the forward pass of deep convolutional neural networks.

In this context, we also highlight [61], which very successfully employs filter learning in convolutional neural networks as regularization prior in image processing tasks. That is, [61] uses simultaneous filter learning and image synthesis for regularization, without prior training. The underlying architecture is strongly related to the energy minimization approach employed here, and again we believe that a deeper mathematical analysis of the latter will be beneficial to explain the success of the former.

Another direct relation to deep neural networks is given via deconvolutional neural networks as discussed in [65], which solve a hierarchy of convolutional sparse coding problems to obtain a feature representation of given image data. Last but not least, we also highlight that the approach discussed in this paper can be employed as feature encoder (again potentially also using incomplete/indirect data measurements), which provides a possible preprocessing step that is very relevant in the context of deep neural networks.

### Outline of the Paper

In Sect. 2, we present the main ideas for our approach in a formal setting. This is done from two perspectives, once from the perspective of a convolutional Lasso approach and once from the perspective of patch-based methods. In Sect. 3, we then carry out an analysis of the proposed model in function space, where we motivate our approach via convex relaxation and derive well-posedness results. Section 4 then presents the model in a discrete setting and the numerical solution strategy, and Sect. 5 provides numerical results and a comparison to the existing methods. At last, an “Appendix” provides a brief overview on some results for tensor spaces that are used in Sect. 3. We note that, while the analysis of Sect. 3 is an important part of our work, the paper is structured in a way such that readers only interested in the conceptual idea and the realization of our approach can skip Sect. 3 and focus on Sects. 2 and 4.

**Fig. 1:**
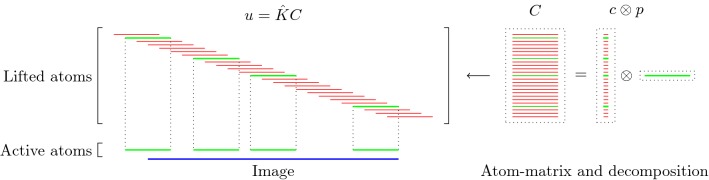
Visualization of the atom-lifting approach for 1D images. The green (thick) lines in the atom matrix correspond to nonzero (active) atoms and are placed in the image at the corresponding positions

## A Convex Approach to Image Atoms

In this section, we present the proposed approach to image-atom-learning and texture reconstruction, where we focus on explaining the main ideas rather than precise definitions of the involved terms. For the latter, we refer to Sect. 3 for the continuous model and Sect. 4 for the discrete setting.

Our starting point is the convolutional Lasso problem [18, 65], which aims to decompose a given image *u* as a sparse linear combination of basic atoms $(p_i)_{i=1}^k$ with coefficient images $(c_i)_{i=1}^k$ by inverting a sum of convolutions as follows

$$\begin{aligned} \min _{ (c_i)_i, (p_i)_i } \sum _{i=1}^k \Vert c_i\Vert _1 \qquad \text {s.t. } {\left\{ \begin{array}{ll} u = \sum _{i=1}^k c_i * p_i, \\ \Vert p_i\Vert _2 \le 1 \text { for } i=1,\ldots ,k. \end{array}\right. } \end{aligned}$$

It is important to note that, by choosing the $(c_i)_i$ to be composed of delta peaks, this allows to place the atoms $(p_i)_i$ at any position in the image. In [65], this model was used in the context of convolutional neural networks for generating image atoms and other image-related tasks. Subsequently, many works have dealt with the algorithmic solution of the resulting optimization problem, where the main difficulty lies in the non-convexity of the atom-learning step, and we refer to [28] for a recent review.

Our goal is to obtain a convex relaxation of this model that can be used for both, learning image atoms from potentially noisy data and image reconstruction tasks such as inpainting, deblurring or denoising. To this aim, we lift the model to the tensor product space of coefficient images and image atoms, i.e., the space of all tensors $C = \sum _{i} c_i \otimes p_i$ with $c_i \otimes p_i$ being a rank-1 tensor such that $(c_i \otimes p_i)(x,y) = c_i(x)p_i(y)$. We refer to Fig. 1 for a visualization of this lifting in a one-dimensional setting, where both coefficients and image atoms are vectors and $c_i \otimes p_i$ corresponds to a rank-one matrix. Notably, in this tensor product space, the convolution $c_i *p_i$ can be written as linear operator ${\hat{K}}$ such that ${\hat{K}}C(x) = \sum _{i} {\hat{K}}(c_i \otimes p_i)(x) = \sum _i \int p_i(x-y)c_i(y) \,\mathrm {d}y$. Exploiting redundancies in the penalization of $(\Vert c_i\Vert _1)_i$ and the constraint $\Vert p_i\Vert _2 \le 1$, $i=1\ldots ,k$ and rewriting the above setting in the lifted tensor space, as discussed in Sect. 3, we obtain the following minimization problem as convex relaxation of the convolutional Lasso approach

$$\begin{aligned} \min _{ C } \Vert C\Vert _{1,2} \qquad \text {s.t. }u = {\hat{K}}C, \end{aligned}$$

where $\Vert \cdot \Vert _{1,2}$ takes the 1-norm and 2-norm of *C* in coefficient and atom direction, respectively. Now while a main feature of the original model was that the number of image atoms was fixed, this is no longer the case in the convex relaxation and would correspond to constraining the rank of the lifted variable *C* (defined as the minimal number of simple tensors needed to decompose *C*) to be below a fixed number. As convex surrogate, we add an additional penalization of the nuclear norm of *C* in the above objective functional (here we refer to the nuclear norm of *C* in the tensor product space which, in the discretization of our setting, coincides with the classical nuclear norm of a matrix reshaping of *C*). Allowing also for additional linear constraints on *C* via a linear operator ${\hat{M}}$, we arrive at the following convex norm that measures the decomposability of a given image *u* into a sparse combination of atoms as

$$\begin{aligned} \begin{aligned}&\mathcal {N} _\nu (u)= {} \min _{C}\, \nu \Vert C\Vert _{1,2 } + (1-\nu )\Vert C\Vert _* \\&{\text {s.t.}}\left\{ \begin{aligned}&u= {\hat{K}}C,\\&{\hat{M}}C = 0. \end{aligned}\right. \end{aligned} \end{aligned}$$

Interestingly, this provides a convex model for learning image atoms, which for simple images admitting a sparse representation seems quite effective. In addition, this can in principle also be used as a prior for image reconstruction tasks in the context of inverse problems via solving for example

$$\begin{aligned} \min _u \frac{\lambda }{2}\Vert Au-u_0\Vert _2^2 + \mathcal {N} _\nu (u) , \end{aligned}$$

with $u_0$ given some corrupted data, *A* a forward operator and $\lambda >0$ a parameter.

Both the original motivation for our model and its convex variant have many similarities with dictionary learning and patch-based methods. The next section strives to clarify similarities and difference and provides a rather interesting, different perspective on our model.

### A Dictionary-Learning-/Patch-Based Methods’ Perspective

In classical dictionary-learning-based approaches, the aim is to represent a resorted matrix of image patches as a sparse combination of dictionary atoms. That is, with $u \in \mathbb {R}^{NM}$ a vectorized version of an image and $D = (D_1,\ldots ,D_l)^T \in \mathbb {R}^{l \times nm}$ a patch matrix containing *l* vectorized (typically overlapping) images patches of size *nm*, the goal is to obtain a decomposition $D = cp$, where $c \in \mathbb {R}^{l \times k}$ is a coefficient matrix and $p \in \mathbb {R}^{k \times nm}$ is a matrix of *k* dictionary atoms such that $c_{i,j}$ is the coefficient for the atom $p_{j,\cdot }$ in the representation of the patch $D_{i}$. In order to achieve a decomposition in this form, using only a sparse representation of dictionary atoms, a classical approach is to solve

$$\begin{aligned} \min _{c,p} \frac{\lambda }{2}\Vert cp - D \Vert _2^2 + \Vert c\Vert _1 \quad \text {s.t. } p \in \mathcal {C} , \end{aligned}$$

where $\mathcal {C}$ potentially puts additional constraints on the dictionary atoms, e.g., ensures that $\Vert p_{j,\cdot } \Vert _2 \le 1$ for all *j*.

**Fig. 2:**
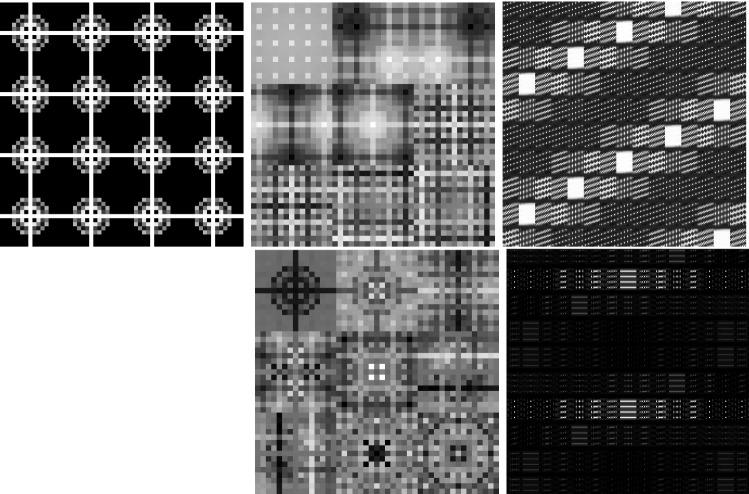
Patch-based representation of test images. Left: original image, middle: nine most important patches for each method (top: patch denoising, bottom: patch reconstruction), right: section of the corresponding patch matrices

A difficulty with such an approach is again the bilinear and hence non-convex nature of the optimization problem, leading to potentially many non-optimal stationary points and making the approach sensitive to initialization.

As a remedy, one strategy is to consider a convex variant (see, for instance, [6]). That is, rewriting the above minimization problem (and again using the ambiguity in the product *cp* to eliminate the $$L^2 $$ constraint) we arrive at the problem

$$\begin{aligned} \min _{C: \text {rank}(C) \le k} \frac{\lambda }{2}\Vert C - D \Vert _2^2 + \Vert C\Vert _{1,2}, \end{aligned}$$

where $\Vert C \Vert _{1,2} = \sum _{i} \Vert C_{i,\cdot } \Vert _2$. A possible convexification is then given as

2 $$\begin{aligned} \min _{C} \frac{\lambda }{2}\Vert C - D \Vert _2^2 + \nu \Vert C\Vert _{1,2} + (1-\nu )\Vert C\Vert _{*}, \end{aligned}$$

where $\Vert \cdot \Vert _{*}$ is the nuclear norm of the matrix *C*.

A disadvantage of such an approach is that the selection of patches is a priori fixed and that the lifted matrix *C* has to approximate each patch. In the situation of overlapping patches, this means that individual rows of *C* have to represent different shifted versions of the same patch several times, which inherently contradicts the low-rank assumption.

It is now interesting to see our proposed approach in relation to these dictionary learning methods and the above-described disadvantage: Denote again by ${\hat{K}}$ the lifted version of the convolution operator, which in the discrete setting takes a lifted patch matrix as input and provides an image composed of overlapping patches as output. It is then easy to see that ${\hat{K}}^*$, the adjoint of ${\hat{K}}$, is in fact a patch selection operator and it holds that ${\hat{K}} {\hat{K}}^* = I$. Now using ${\hat{K}}^*$, the approach in (2) can be rewritten as

3 $$\begin{aligned} \min _{C} \frac{\lambda }{2}\Vert C - {\hat{K}}^*u \Vert _2^2 + \nu \Vert C\Vert _{1,2} + (1-\nu )\Vert C\Vert _{*}, \end{aligned}$$

where we remember that *u* is the original image. Considering the problem of finding an optimal patch-based representation of an image as the problem of inverting ${\hat{K}}$, we can see that the previous approach in fact first applies a right inverse of ${\hat{K}}$ and then decomposes the data. Taking this perspective, however, it seems much more natural to consider instead an adjoint formulation as

4 $$\begin{aligned} \min _{C} \frac{\lambda }{2}\Vert {\hat{K}}C - u \Vert _2^2 + \nu \Vert C\Vert _{1,2} + (1-\nu )\Vert C\Vert _{*}. \end{aligned}$$

Indeed, this means that we do not fix the patch decomposition of the image a priori but rather allow the method itself to optimally select the position and size of patches. In particular, given a particular patch at an arbitrary location, this patch can be represented by using only one line of *C* and the other lines (corresponding to shifted versions) can be left empty. Figure 2 shows the resulting improvement by solving both of the above optimization problems for a particular test image, where the parameters are set such that the data error of both methods, i.e., $\Vert {\hat{K}}C -u \Vert _2 ^2$, is approximately the same. As can be seen, solving (3), which we call patch denoising, does not yield meaningful dictionary atoms as the dictionary elements need to represent different, shifted version of the single patch that makes up the image. In contrast to that, solving (4), which we call patch reconstruction, allows to identify the underlying patch of the image and the corresponding patch matrix is indeed row sparse. In this context, we also refer to [26] which makes similar observations and differs between patch analysis (which is related to (3)) and patch synthesis, which is similar to (4); however, it does not consider a convolutional- but rather a matrix-based synthesis operator.

### The Variational Model

Now while the proposed model can, in principle, describe any kind of image, in particular its convex relaxation seems best suited for situations where the image can be described by only a few, repeating atoms, as would be, for instance, the case with texture images. In particular, since we do not include rotations in the model, there are many simple situations, such as *u* being the characteristic function of a disk, which would in fact require an infinite number of atoms. To overcome this, it seems beneficial to include an additional term which is rotationally invariant and takes care of piecewise smooth parts of the image. Denoting $\mathcal {R}$ to be any regularization functional for piecewise smooth data and taking the infimal convolution of this functional with our atom-based norm, we then arrive at the convex model

$$\begin{aligned} \min _{u,v} \frac{\lambda }{2}\Vert Au-u_0\Vert _2^2 + \mu _1 \mathcal {R} (u-v) + \mu _2 \mathcal {N} _\nu (v), \end{aligned}$$

for learning image data and image atom kernels from potentially noisy or incomplete measurements.

A particular example of this model can be given when choosing $\mathcal {R} ={{\,\mathrm{TV}\,}}$, the total variation function [51]. In this setting, a natural choice for the operator *M* in the definition of $\mathcal {N} _\nu$ is to take the pointwise mean of the lifted variable in atom direction, which corresponds to constraining the learned atoms to have zero mean and enforces some kind of orthogonality between the cartoon and the texture part in the spirit of [43]. In our numerical experiments, in order to obtain an even richer model for the cartoon part, we use the second-order total generalized variation function (${{\,\mathrm{TGV}\,}}_\alpha ^2$) [7, 9] as cartoon prior and, in the spirit of a dual ${{\,\mathrm{TGV}\,}}_\alpha ^2$ norm, use *M* to constrain the 0th and 1st moments of the atoms to be zero.

We also remark that, as shown in the analysis part of Sect. 3, while an $\ell ^1/\ell ^2$-type norm on the lifted variables indeed arises as convex relaxation of the convolutional Lasso approach, the addition of the nuclear norm is to some extent arbitrary and in fact, in the context of compressed sensing, it is known that a summation of two norms is suboptimal for a joint penalization of sparsity and rank [44]. (We refer to Remark 5 for an extended discussion.) Indeed, our numerical experiments also indicate that the performance of our method is to some extent limited by a suboptimal relaxation of a joint sparsity and rank penalization. To account for that, we also tested with semi-convex potential functions (instead of the identity) for a penalization of the singular values in the nuclear norm. Since this provided a significant improvement in some situations, we also include this more general setting in our analysis and the numerical results.

## The Model in a Continuous Setting

The goal of this section is to define and analyze the model introduced in Sect. 2 in a continuous setting. To this aim, we regard images as functions in the Lebesgue space $L^q(\Omega )$ with a bounded Lipschitz domain $\Omega \subset \mathbb {R}^d$, $d \in \mathbb {N}$ and $1 < q \le 2$. Image atoms are regarded as functions in $L^s(\Sigma )$, with $\Sigma \subset \mathbb {R}^d$ a second (smaller) bounded Lipschitz domain (either a circle or a square around the origin) and $s \in [q,\infty ]$ an exponent that is a priori allowed to take any value in $[q,\infty ]$, but will be further restricted below. We also refer to “Appendix” for further notation and results, in particular in the context of tensor product spaces, that will be used in this section.

As described in Sect. 2, the main idea is to synthesize an image via the convolution of a small number of atoms with corresponding coefficient images, where we think of the latter as a sum of delta peaks that define the locations where atoms are placed. For this reason, and also due to compactness properties, the coefficient images are modeled as Radon measures in the space $\mathcal {M}(\Omega _\Sigma )$, the dual of $C_0(\Omega _\Sigma )$, where we denote

$$\begin{aligned} \Omega _\Sigma : = \{ x \in \mathbb {R}^d \, \left| \right. \, \text {there exists }y \in \Sigma \text { s.t. } x-y \in \Omega \}, \end{aligned}$$

i.e., the extension of $\Omega$ by $\Sigma$. The motivation for using this extension of $\Omega$ is to allow atoms also to be placed arbitrarily close to be boundary (see Fig. 1). We will further use the notation $r'=r/(r-1)$ for an exponent $r \in (1,\infty )$ and denote duality pairings between $L^r$ and $L^{r'}$ and between $\mathcal {M}(\Omega )$ and $C_0(\Omega )$ by $(\cdot ,\cdot )$, while other duality pairings (e.g., between tensor spaces) are denoted by $\langle \cdot ,\cdot \rangle$. By $\Vert \cdot \Vert _{r}, \Vert \cdot \Vert _\mathcal {M}$, we denote standard $L^r$ and Radon norms whenever the domain of definition is clear from the context, otherwise we write $\Vert \cdot \Vert _{L^r(\Omega _\Sigma )},\Vert \cdot \Vert _{\mathcal {M}(\Omega _\Sigma )}$, etc.

### The Convolutional Lasso Prior

As a first step, we deal with the convolution operator that synthesizes an image from a pair of a coefficient image and an image atom in function space. Formally, we aim to define $K:\mathcal {M}(\Omega _\Sigma ) \times L^s(\Sigma ) \rightarrow L^q(\Omega )$ as

$$\begin{aligned} K(c,p)(x): = \int _{\Omega _\Sigma } p(x-y) \,\mathrm {d}c(y), \end{aligned}$$

where we extend *p* by zero outside of $\Sigma$. An issue with this definition is that, in general, *p* is only defined Lebesgue almost everywhere and so we have to give a rigorous meaning to the integration of *p* with respect to an arbitrary Radon measure. To this aim, we define the convolution operator via duality (see [52]). For $c \in \mathcal {M}(\Omega _\Sigma )$, $p \in L^s(\Sigma )$ we define by $K_{c,p}$ the functional on $C({\overline{\Omega }})$ as dense subset of $L^{q'}(\Omega )$ as

$$\begin{aligned} K_{c,p}(h):= \int _{\mathbb {R}^d} \int _{\mathbb {R}^d} {\tilde{h}}(z+y) {\tilde{p}}(z) \,\mathrm {d}z \,\mathrm {d}{\tilde{c}}(y), \end{aligned}$$

where ${\tilde{g}}$ always denotes the zero extension of the function or measure *g* outside their domain of definition. Now we can estimate with $\Theta >0$

$$\begin{aligned} \left| K_{c,p}(h) \right|\le & {} \int _{\mathbb {R}^d} \int _{\mathbb {R}^d} |{\tilde{h}}(z+y)| |{\tilde{p}}(z)| \,\mathrm {d}z \,\mathrm {d}|{\tilde{c}}|(y)\\\le & {} \Vert {\tilde{h}}\Vert _{L^{q'}(\mathbb {R}^d)} \Vert {\tilde{p}}\Vert _{L^{q}(\mathbb {R}^d)} \Vert {\tilde{c}}\Vert _{\mathcal {M}(\mathbb {R}^d)}\\\le & {} \Theta \Vert p\Vert _{s} \Vert c\Vert _{\mathcal {M}} \Vert h\Vert _{q'}. \end{aligned}$$

Hence, by density we can uniquely extend $K_{c,p}$ to a functional in $L^{q'}(\Omega )^*\simeq L^q(\Omega )$ and we denote by $[K_{c,p}]$ the associated function in $L^q(\Omega )$. Now in case *p* is integrable w.r.t. *c* and $x \mapsto \int _{\Omega _\Sigma } p(x-y) \,\mathrm {d}c(y) \in L^q(\Omega )$, we get by a change of variables and Fubini’s theorem that for any $h\in C({\overline{\Omega }})$

$$\begin{aligned} K_{c,p}(h)= & {} \int _{\mathbb {R}^d} \int _{\mathbb {R}^d} {\tilde{h}}(x) {\tilde{p}}(x-y) \,\mathrm {d}x \,\mathrm {d}{\tilde{c}}(y) \\= & {} \int _\Omega h(x)\left( \int _{\Omega _\Sigma } p(x-y) \,\mathrm {d}c(y) \right) \,\mathrm {d}x. \end{aligned}$$

Hence we get that in this case, $[K_{c,p}](x) = \int _{\Omega _\Sigma } p(x-y) \,\mathrm {d}c(y)$ and defining $K:\mathcal {M}(\Omega _\Sigma ) \times L^s(\Omega ) \rightarrow L^q(\Omega )$ as

$$\begin{aligned} K(c,p):= [K_{c,p}] \end{aligned}$$

we get that *K*(*c*, *p*) coincides with the convolution of *c* and *p* whenever the latter is well defined. Note that *K* is bilinear and, as the previous estimate shows, there exists $\Theta >0$ such that $\Vert K(c,p)\Vert _q \le \Theta \Vert c\Vert _\mathcal {M}\Vert p\Vert _s$. Hence, $K \in \mathcal {B} (\mathcal {M}(\Omega _\Sigma ) \times L^s(\Sigma ),L^q(\Omega ))$, the space of bounded bilinear operators (see “Appendix”).

Using the bilinear operator *K* and denoting by $k\in \mathbb {N}$ a fixed number of atoms, we now define the convolutional Lasso prior for an exponent $s \in [q,\infty ]$ and for $u \in L^q(\Omega )$ as

5 $$\begin{aligned} \begin{aligned}&\mathcal {N}_\text {cl,s}(u) = \inf _ { \begin{array}{c} (c_i)_{i=1}^k \subset \mathcal {M}(\Omega _\Sigma ) \\ (p_i)_{i=1}^k \subset L^s (\Sigma ) \end{array}} \, \sum _{i=1}^k \Vert c_i\Vert _\mathcal {M}\\&\quad \text {s.t. } \left\{ \begin{aligned}&\Vert p_i\Vert _s \le 1,\, Mp_i = 0 \quad i=1,\ldots ,k, \\&u = \sum _{i=1}^k K(c_i,p_i) \text { in } \Omega , \end{aligned} \right. \end{aligned} \end{aligned}$$

and set $\mathcal {N}_\text {cl,s}(u) = \infty$ if the constraint set above is empty. Here, we include an operator $M \in \mathcal {L}(L^s(\Sigma ),\mathbb {R}^m)$ in our model that optionally allows to enforce additional constraints on the atoms. A simple example of *M* that we have in mind is an averaging operator, i.e., ${\text {Mp}}:= |\Sigma |^{-1} \int _\Sigma p(x) \,\mathrm {d}x$; hence, the constraint that ${\text {Mp}} = 0$ corresponds to a zero-mean constraint.

### A Convex Relaxation

Our goal is now to obtain a convex relaxation of the convolutional Lasso prior. To this aim, we introduce by ${\hat{K}}$ and ${\hat{M}}:=I \otimes M$ the lifting of the bilinear operator *K* and the linear operators *I* and *M*, with $I\in \mathcal {L}(\mathcal {M}(\Omega _\Sigma ),\mathcal {M}(\Omega _\Sigma ))$ being the identity, to the projective tensor product space $X_s:=\mathcal {M}(\Omega _\Sigma )\otimes _\pi L^s(\Sigma )$ (see “Appendix”). In this space, we consider a reformulation as

6 $$\begin{aligned} \begin{aligned} \mathcal {N}_\text {cl,s}(u) = \inf _ {C \in X_s} \, \Vert C\Vert _{ \pi ,k,M}\quad \text {s.t. } \begin{aligned}&u = {\hat{K}}C \text { in } \Omega , \end{aligned} \end{aligned} \end{aligned}$$

where

$$\begin{aligned}&\Vert C\Vert _{ \pi ,k,M} := \inf \left\{ \sum _{i=1}^k \Vert c_i\Vert _\mathcal {M}\Vert p_i\Vert _s \, \left| \right. \, C \right. \\&\quad \left. = \sum _{i=1}^k c_i \otimes p_i \text { with } {\text {Mp}}_i = 0 \text { for } i=1,\ldots ,k \right\} . \end{aligned}$$

Note that this reformulation is indeed equivalent. Next we aim to derive the convex relaxation of $$\mathcal {N}_\text {cl,s}$$ in this tensor product space. To this aim, we use the fact that for a general function $$g:X_s \rightarrow {\overline{\mathbb {R}}}$$, its convex, lower semi-continuous relaxation can be computed as the biconjugate $$g^{**}:X_s \rightarrow {\overline{\mathbb {R}}}$$, where $$g^*(x^*) = \sup _{x\in X_s} \langle x^*,x\rangle - g(x)$$ and $$g^{**}(x) = \sup _{x^*\in X^*_s} \langle x^*,x\rangle - g^*(x^*)$$.

First we consider a relaxation of the functional $\Vert \cdot \Vert _{ \pi ,k,M}$. In this context, we need an additional assumption on the constraint set $\ker (M)$, which is satisfied, for instance, if $s=2$ or for $M=0$, in particular will be fulfilled by the concrete setting we use later on.

#### Lemma 1

Assume that there exists a continuous, linear, norm-one projection onto $\ker (M)$. Then, the convex, lower semi-continuous relaxation of $\Vert \cdot \Vert _{\pi ,k,M}:X_s \rightarrow {\overline{\mathbb {R}}}$ is given as

$$\begin{aligned} C \mapsto \Vert C\Vert _{\pi } + \mathcal {I}_{\ker ({\hat{M}})}(C), \end{aligned}$$

where $\mathcal {I}_{\ker ({\hat{M}})}(C)= 0$ if ${\hat{M}}C = 0$ and $\mathcal {I}_{\ker ({\hat{M}})}(C)= \infty$ else, and $\Vert \cdot \Vert _{\pi }$ is the projective norm on $X_s$ given as

$$\begin{aligned} \Vert C\Vert _\pi = \inf \left\{ \sum _{i=1}^\infty \Vert c_i\Vert _\mathcal {M}\Vert p_i\Vert _s \, \left| \right. \, C = \sum _{i=1}^\infty c_i \otimes p_i \right\} . \end{aligned}$$

#### Proof

Our goal is to compute the biconjugate of $\Vert \cdot \Vert _{\pi ,k,M}$. First we note that

$$\begin{aligned} \Vert \cdot \Vert _{\pi } + \mathcal {I}_{\ker ({\hat{M}})} \le \Vert \cdot \Vert _{\pi ,k,M}\le \Vert \cdot \Vert _{\pi ,1,M} , \end{aligned}$$

and consequently

$$\begin{aligned} \Vert \cdot \Vert _{\pi } + \mathcal {I}_{\ker ({\hat{M}})} \le \left( \Vert \cdot \Vert _{\pi ,k,M} \right) ^{**} \le \left( \Vert \cdot \Vert _{\pi ,1,M} \right) ^{**}. \end{aligned}$$

Hence, the assertion follows if we show that $$\left( \Vert \cdot \Vert _{\pi ,1,M} \right) ^{**} \le \Vert \cdot \Vert _{\pi } + \mathcal {I}_{\ker ({\hat{M}})}.$$ To this aim, we first show that $$\Vert \cdot \Vert _{\pi ,M} \le \Vert \cdot \Vert _{\pi } + \mathcal {I}_{\ker ({\hat{M}})} $$, where we set $$\Vert C\Vert _{ \pi ,M} = \Vert C\Vert _{ \pi ,\infty ,M}$$. Let $$C \in X_s$$ be such that $${\hat{M}}C=0$$ and take $$(c_i)_i$$, $$(p_i)_i$$ be such that $$\Vert C\Vert _\pi \ge \sum _{i=1}^\infty \Vert c_i\Vert _\mathcal {M}\Vert p_i\Vert _s - \epsilon $$ for some $$\epsilon >0$$ and $$C = \sum _{i=1}^\infty c_i \otimes p_i$$. Then, with *P* the projection to $$\ker (M)$$ as in the assumption, we get that

$$\begin{aligned} 0 = {\hat{M}}C = \sum _{i=1}^\infty c_i \otimes M p_i = \sum _{i=1}^\infty c_i \otimes M (p_i - Pp_i). \end{aligned}$$

Now remember that, according to [53, Theorem 2.9], we have $(\mathcal {M}(\Omega _\Sigma ) \otimes _\pi \mathbb {R}^m)^* = {\mathcal {B}}(\mathcal {M}(\Omega _\Sigma ) \times \mathbb {R}^m)$ with the norm $\Vert B\Vert _{{\mathcal {B}}}:= \sup \{ |B(x,y)| \, \left| \right. \, \Vert x\Vert _\mathcal {M}\le 1, \, \Vert y\Vert _2 \le 1 \}$. Taking arbitrary $\psi \in \mathcal {M}(\Omega )^*$, $\phi \in (\mathbb {R}^m)^*$, we get that $B:(c,p) \mapsto \psi (c)\phi (p) \in {\mathcal {B}}(\mathcal {M}(\Omega _\Sigma ) \times \mathbb {R}^m)$ and hence

$$\begin{aligned} 0&= {\hat{B}}({\hat{M}}C) \\&= \sum _{i=1}^\infty {\hat{B}}(c_i \otimes M (p_i - Pp_i))\\&= \sum _{i=1}^\infty \psi (c_i) \phi ( M (p_i - Pp_i)) \\&= \phi \left( \sum _{i=1}^\infty \psi (c_i) M (p_i - Pp_i) \right) \\&= \phi \left( M \left( \sum _{i=1}^\infty \psi (c_i) (p_i - Pp_i) \right) \right) \end{aligned}$$

and since $\phi$ was arbitrary, it follows that $M ( \sum _{i=1}^\infty \psi (c_i) (p_i - Pp_i) ) = 0$. Finally, by closedness of ${{\,\mathrm{Rg}\,}}(I-P)$ we get that $\sum _{i=1}^\infty \psi (c_i) (p_i - Pp_i) = 0$ and, since $\mathcal {M}(\Omega _\Sigma )$ has the approximation property (see [24, Section VIII.3]), from [53, Proposition 4.6], it follows that $\sum _{i=1}^\infty c_i \otimes (p_i - Pp_i) = 0$, hence $C = \sum _{i=1}^\infty c_i \otimes Pp_i$ and by assumption $\sum _{i=1}^\infty \Vert c_i\Vert _\mathcal {M}\Vert p_i\Vert _s \ge \sum _{i=1}^\infty \Vert c_i\Vert _\mathcal {M}\Vert Pp_i\Vert _s$. Consequently, $\Vert \cdot \Vert _{\pi } + \mathcal {I}_{\ker ({\hat{M}})} \ge \Vert \cdot \Vert _{\pi ,M} - \epsilon$, and since $\epsilon$ was arbitrary, the claimed inequality follows.

Now we show that $\left( \Vert \cdot \Vert _{\pi ,M} \right) ^{*} \le \left( \Vert \cdot \Vert _{\pi ,1,M} \right) ^{*}$, from which the claimed assertion follows by the previous estimate and taking the convex conjugate on both sides. To this aim, take $(C_n)_n \subset X_s$ such that

$$\begin{aligned} \left( \Vert \cdot \Vert _{\pi ,M} \right) ^* (B)= & {} \sup _{C \in X_s} \langle B,C\rangle - \Vert C\Vert _{\pi ,M} \\= & {} \lim _n \langle B,C_n\rangle - \Vert C_n\Vert _{\pi ,M} \end{aligned}$$

and take $(c_i^n)_i$, $(p_i^n)_i$ such that ${\text {Mp}}^n_i=0$ for all *n*, *i* and

$$\begin{aligned} C_n = \sum _{i=1}^\infty c^n_i \otimes p^n_i \quad \text {and}\quad \sum _{i=1}^\infty \Vert c_i^n\Vert _\mathcal {M}\Vert p_i^n\Vert _s \le \Vert C_n\Vert _{\pi ,M} + 1/n . \end{aligned}$$

We then get

$$\begin{aligned} \left( \Vert \cdot \Vert _{\pi ,M} \right) ^* (B)&= \lim _n \langle B,C_n\rangle - \Vert C_n\Vert _{\pi ,M} \le \lim _n \langle B, \sum _{i=1}^\infty c^n_i \otimes p^n_i\rangle \\&\quad - \sum _{i=1}^\infty \Vert c_i^n\Vert _\mathcal {M}\Vert p_i^n\Vert _s + 1/n \\&= \lim _n\lim _m \langle B, \sum _{i=1}^m c^n_i \otimes p^n_i\rangle \\&\quad - \sum _{i=1}^m \Vert c_i^n\Vert _\mathcal {M}\Vert p_i^n\Vert _s + 1/n \\&\le \lim _n \sup _m \sup _{ \begin{array}{c} (c_i)_{i=1}^m ,(p_i)_{i=1}^m \\ {\text {Mp}}_i = 0 \end{array} }\langle B, \sum _{i=1}^m c_i \otimes p_i\rangle \\&\quad - \sum _{i=1}^m \Vert c_i\Vert _\mathcal {M}\Vert p_i\Vert _s + 1/n \\&= \sup _m \sup _{\begin{array}{c} (c_i)_{i=1}^m ,(p_i)_{i=1}^m \\ {\text {Mp}}_i = 0 \end{array}} \sum _{i=1}^m B(c_i,p_i) - \Vert c_i\Vert _\mathcal {M}\Vert p_i\Vert _s \\&= \sup _m m \sup _{\begin{array}{c} c,p \\ {\text {Mp}} = 0 \end{array}} \left( B(c,p) - \Vert c\Vert _\mathcal {M}\Vert p\Vert _s \right) . \end{aligned}$$

Now it can be easily seen that the last expression equals 0 in case $|B(c,p)| \le \Vert c\Vert _\mathcal {M}\Vert p\Vert _s$ for all *c*, *p* with ${\text {Mp}} = 0$. In the other case, we can pick ${\hat{c}},{\hat{p}}$ with $M{\hat{p}}=0$ and $\Theta >1$ such that $B({\hat{c}},{\hat{p}}) > \Theta \Vert {\hat{c}}\Vert _\mathcal {M}\Vert {\hat{p}}\Vert _s$ and get for any $\lambda >0$ that

$$\begin{aligned}&\sup _{\begin{array}{c} c,p \\ {\text {Mp}} = 0 \end{array}} B(c,p) - \Vert c\Vert _\mathcal {M}\Vert p\Vert _s \\&\quad \ge B(\lambda {\hat{c}},{\hat{p}}) - \Vert \lambda {\hat{c}}\Vert _\mathcal {M}\Vert {\hat{p}}\Vert _s \ge \lambda (\Theta -1) \rightarrow \infty \text { as }\lambda \rightarrow \infty . \end{aligned}$$

Hence, the last line of the above equation is either 0 or infinity and equals

$$\begin{aligned} \sup _{\begin{array}{c} c,p \\ {\text {Mp}} = 0 \end{array}} \left( B(c,p) - \Vert c\Vert _\mathcal {M}\Vert p\Vert _s \right)= & {} \sup _C \langle B,C \rangle - \Vert C\Vert _{\pi ,1,M} \\= & {} \left( \Vert \cdot \Vert _{\pi ,1,M} \right) ^*. \end{aligned}$$

$$\square $$

This result suggests that the convex, lower semi-continuous relaxation of (6) will be obtained by replacing $\Vert \cdot \Vert _{\pi ,k,M}$ with the projective tensor norm $\Vert \cdot \Vert _{\pi }$ on $X_s$ and the constraint ${\hat{M}}C = 0$. Our approach to show this will in particular require us to ensure lower semi-continuity of this candidate for the relaxation, which in turn requires us to ensure a compactness property of the sublevel sets of the energy appearing in (6) and closedness of the constraints. To this aim, we consider a weak* topology on $X_s$ and rely on a duality result for tensor product spaces (see “Appendix”), which states that, under some conditions, the projective tensor product $X_s = \mathcal {M}(\Omega _\Sigma ) \otimes _\pi L^s(\Sigma )$ can be identified with the dual of the so-called injective tensor product $C_0(\Omega _\Sigma ) \otimes _\mathfrak {i}L^{s'}(\Sigma )$. The weak* topology on $X_s$ is then induced by pointwise convergence in $X_s$ as dual space of $C_0(\Omega _\Sigma ) \otimes _\mathfrak {i}L^{s'}(\Sigma )$. Different from what one would expect from the individual spaces, however, this can only be ensured for the case $s < \infty$ which excludes the space $L^\infty (\Sigma )$ for the image atoms. This restriction will also be required later on in order to show well-posedness of a resulting regularization approach for inverse problems, and hence, we will henceforth always consider the case that $s \in [q,\infty )$ and use the identification $(C_0(\Omega _\Sigma ) \otimes _\mathfrak {i}L^{s'}(\Sigma ))^* \hat{=}\mathcal {M}(\Omega _\Sigma ) \otimes _\pi L^s(\Sigma )$ (see “Appendix”).

As a first step toward the final relaxation result, and also as a crucial ingredient for well-posedness results below, we show weak* continuity of the operator ${\hat{K}}$ on the space $X_s$.

#### Lemma 2

Let $$s \in [q,\infty )$$. Then, the operator $${\hat{K}}:X_s \rightarrow L^q(\Omega )$$ is continuous w.r.t. weak* convergence in $$ X_s$$ and weak convergence in $$L^{q'}(\Omega )$$. Also, for any $$\phi \in C_0(\Omega ) \subset L^{q'}(\Omega )$$ it follows that $${\hat{K}}^*\phi \in C_0(\Omega _\Sigma ) \otimes _\mathfrak {i}L^{s'}(\Sigma ) $$ and, via the identification $$C_0(\Omega _\Sigma ) \otimes _\mathfrak {i}L^{s'}(\Sigma ) \hat{=} C_0(\Omega _\Sigma ,L^{s'}(\Sigma ))$$ (see “Appendix”), can be given as $$K^*\phi (t)= [ x \mapsto \phi (t + x) ]$$.

#### Proof

First we note that for any $\psi \in C_c(\Omega )$, the function ${\hat{\psi }}$ defined as ${\hat{\psi }}(t)=[ x \mapsto \psi (t+x)]$ (where we extend $\psi$ by 0 to $\mathbb {R}^d$) is contained in $C_c(\Omega _\Sigma ,L^{s'}(\Sigma ))$. Indeed, continuity follows since by uniform continuity for any $\epsilon > 0$ there exists a $\delta > 0$ such that for any $r\in \mathbb {R}^d$ with $|r| \le \delta$ and $t \in \Omega _\Sigma$ with $t+r\in \Omega _\Sigma$

$$\begin{aligned} \Vert {\hat{\psi }}(t+r)-{\hat{\psi }}(t)\Vert _{s'}= & {} \left( \int _{\Sigma } |\psi (t +r+ x) - \psi (t + x)|^{s'}\,\mathrm {d}x \right) ^{1/{s'}} \\\le & {} \epsilon |\Sigma |^{1/{s'}} . \end{aligned}$$

Also, taking $K \subset \Omega$ to be the support of $\psi$ we get, with $K_\Sigma$ the extension of *K* by $\Sigma$, for any $t \in \Omega _\Sigma \setminus K_\Sigma$ that $\psi (t + x)=0$ for any $x \in \Sigma$ and hence ${\hat{\psi }}=0$ in $L^{s'}(\Sigma )$ and ${\hat{\psi }}\in C_c(\Omega _\Sigma ,L^{s'}(\Sigma ))$.

Now for $\phi \in C_0(\Omega _\Sigma )$, taking $(\phi _n)_n \in C_c (\Omega _\Sigma )$ to be a sequence converging to $\phi$, we get that

$$\begin{aligned} \Vert {\hat{\phi }} - \hat{\phi _n}\Vert _{C_0(\Omega _\Sigma ,L^{s'}(\Sigma )) }= & {} \sup _t \left( \int _\Sigma | \phi (t+x) - \phi _n (t+x) |^{s'} \,\mathrm {d}x \right) ^{1/{s'}}\\\le & {} \Vert \phi - \phi _n \Vert _\infty |\Sigma |^{1/{s'}} \rightarrow 0 . \end{aligned}$$

Thus, ${\hat{\phi }}$ can be approximated by a sequence of compactly supported functions and hence ${\hat{\phi }} \in C_0(\Omega _\Sigma ,L^{s'}(\Sigma ) )$. Fixing now $u =c \otimes p\in X_s$, we note that for any $\psi \in C_0(\Omega _\Sigma ,L^{s'}(\Sigma ))$, the function $t \rightarrow \int _\Sigma \psi (t)(x)p(x)\,\mathrm {d}x$ is continuous; hence, we can define the linear functional

$$\begin{aligned} \mathcal {F} _{u}(\psi ):= \int _{\Omega _\Sigma } \int _\Sigma \psi (t)(x) p(x) \,\mathrm {d}x \,\mathrm {d}c(t) \end{aligned}$$

and get that $\mathcal {F} _u$ is continuous on $C_0(\Omega _\Sigma ,L^{s'}(\Sigma ))$. Then, since ${\hat{\phi }}\in C_0(\Omega _\Sigma ) \otimes _\mathfrak {i}L^{s'}(\Sigma )$ it can be approximated by a sequence of simple tensors $(\sum _{i=1}^{m_n} x_i^n \otimes y_i^n)_n$ in the injective norm, which coincides with the norm in $C_0(\Omega _\Sigma ,L^{s'}(\Sigma ))$ and, using Lemma 22 in “Appendix”, we get

$$\begin{aligned} \langle u,{\hat{\phi }} \rangle&= \lim _n \langle u, \sum _{i=1}^{m_n} x_i^n \otimes y_i^n \rangle = \lim _n \sum _{i=1}^{m_n} (c,x_i^n)(p,y_i^n) \\&= \lim _n \sum _{i=1}^{m_n} \int _{\Omega _\Sigma } \int _\Sigma x_i^n(a)y_i^n(b) p(b)\,\mathrm {d}b\,\mathrm {d}c(a) \\&= \lim _n \mathcal {F} _u \left( \left( \sum _{i=1}^{m_n} x_i^n \otimes y_i^n\right) _n\right) = \mathcal {F} _u ({\hat{\phi }}) \\&= \int _{\Omega _\Sigma } \int _\Sigma \phi (a+b) p(b) \,\mathrm {d}b\,\mathrm {d}c(a) \\&= (K(c , p),\phi ) = ({\hat{K}}u,\phi ) \end{aligned}$$

Now by density of simple tensors in the projective tensor product, it follows that $$K^*\phi = {\hat{\phi }}$$. In order to show the continuity assertion, take $$(u_n)_n $$ weak * converging to some $$u \in X_s$$. Then by the previous assertion we get for any $$\phi \in C_c (\Omega _\Sigma )$$ that

$$\begin{aligned} (Ku_n,\phi ) = \langle u_n,K^* \phi \rangle \rightarrow \langle u,K^*\phi \rangle = (Ku,\phi ) , \end{aligned}$$

hence $(Ku_n)_n$ weakly converges to *Ku* on a dense subset of $L^{s'}(\Omega )$ which, together with boundedness of $(Ku_n)_n$, implies weak convergence. $\square$

We will also need weak*-to-weak* continuity of ${\hat{M}}$, which is shown in the following lemma in a slightly more general situation than needed.

#### Lemma 3

Take $s \in [q,\infty )$ and assume that $M\in \mathcal {L}(L^s(\Sigma ), Z)$ with *Z* a reflexive space and define ${\hat{M}}:=I \otimes _\pi M \in \mathcal {L}(X_s ,\mathcal {M}(\Omega _{\Sigma }) \otimes _\pi Z)$, where *I* is the identity on $\mathcal {M}(\Omega _{\Sigma })$. Then ${\hat{M}}$ is continuous w.r.t. weak* convergence in both spaces.

#### Proof

Take $(u_n)_n \in X$ weak* converging to some $u \in X$ and write $u_n = \lim _k \sum _{i=1}^k x_i^n \otimes y_i^n$. We note that, since *Z* is reflexive, it satisfies in particular the Radon Nikodým property (see “Appendix”) and hence $C(\Omega _{\Sigma }) \otimes _\mathfrak {i}Z^*$ can be regarded as predual of $\mathcal {M}(\Omega _{\Sigma }) \otimes _\pi Z$ and we test with $\phi \otimes \psi \in C(\Omega _{\Sigma }) \otimes _\mathfrak {i}Z^*$. Then

$$\begin{aligned} \langle {\hat{M}}u_n,\phi \otimes \psi \rangle&= \lim _k \sum _{i=1}^k ( x_i^n,\phi ) ( My_i^n,\psi )\\&= \lim _k \sum _{i=1}^k ( x_i^n,\phi ) ( y_i^n,M^*\psi ) \\&= \langle u_n , \phi \otimes M^* \psi \rangle \rightarrow \langle u , \phi \otimes M^* \psi \rangle \\&= \langle {\hat{M}}u,\phi \otimes \psi \rangle , \end{aligned}$$

where the convergence follows since $$M^* \psi \in L^{s'}(\Sigma )$$, the predual of $$L^s(\Sigma )$$, and hence, $$\phi \otimes M^* \psi \in C(\Omega _{\Sigma }) \otimes _\mathfrak {i}L^{q'}(\Sigma ) .$$$$\square $$

Now we can obtain the convex, lower semi-continuous relaxation of $\mathcal {N}_\text {cl,s}$.

#### Lemma 4

With the assumptions of Lemma 1 and $s \in [q,\infty )$, the convex, l.s.c. relaxation of $\mathcal {N}_\text {cl,s}$ is given as

7 $$\begin{aligned} \mathcal {N}_\text {cl,s}^{**}(u) = \inf _{C \in X_s} \Vert C\Vert _\pi \quad \text {s.t. } {\left\{ \begin{array}{ll} u = {\hat{K}}C \text { in }\Omega , \\ {\hat{M}}C = 0. \end{array}\right. } \end{aligned}$$

#### Proof

Again we first compute the convex conjugate:

$$\begin{aligned} \mathcal {N}_\text {cl,s}^* (v)&= \sup _u (u,v) - \mathcal {N}_\text {cl,s}(u) = \sup _{ C \in X_s } ({\hat{K}}C,v) - \Vert C\Vert _{\pi ,k,M} \\&= \sup _{ C \in X_s } \langle C,{\hat{K}}^*v\rangle - \Vert C\Vert _{\pi ,k,M} = \Vert {\hat{K}}^ * v \Vert _{\pi ,k,M}^*. \end{aligned}$$

Similarly, we see that $\mathcal {N} ^*(v) = \left( \Vert \cdot \Vert _\pi + \mathcal {I}_{\ker ({\hat{M}})} \right) ^* ({\hat{K}}^* v)$, where

$$\begin{aligned} \mathcal {N} (u) = \inf _{C \in X_s} \Vert C\Vert _\pi \quad \text {s.t. } {\left\{ \begin{array}{ll} u = {\hat{K}}C \text { in }\Omega , \\ {\hat{M}}C = 0. \end{array}\right. } \end{aligned}$$

Now in the proof of Lemma 1, we have in particularly shown that $$ \left( \Vert \cdot \Vert _\pi + \mathcal {I}_{\ker ({\hat{M}})} \right) ^* = \Vert \cdot \Vert _{\pi ,k,M}^*$$; hence, if we show that $$ \mathcal {N} $$ is convex and lower semi-continuous, the assertion follows from $$ \mathcal {N} (u) = \mathcal {N} ^{**}(u) = \mathcal {N}_\text {cl,s}^{**}(u)$$. To this aim, take a sequence $$(u_n)_n $$ in $$L^q(\Omega )$$ converging weakly to some *u* for which, without loss of generality, we assume that

$$\begin{aligned} \lim _n \mathcal {N} (u_n) = \liminf _n \mathcal {N} (u_n) < \infty . \end{aligned}$$

Now with $(C_n)_n$ such that $\Vert C_n\Vert _\pi \le \mathcal {N} (u_n) +n^{-1}$, ${\hat{M}}C_n = 0$ and $u_n = {\hat{K}}C_n$ we get that $(\Vert C_n\Vert _\pi )_n$ is bounded. Since $X_s$ admits a separable predual (see “Appendix”), this implies that $(C_n)_n$ admits a subsequence $(C_{n_i})_i$ weak* converging to some *C*. By weak* continuity of ${\hat{K}}$ and ${\hat{M}}$ we get that $u = {\hat{K}}C$ and ${\hat{M}}C=0$, respectively, and by weak* lower semi-continuity of $\Vert \cdot \Vert _\pi$, it follows that

$$\begin{aligned} \mathcal {N} (u)\le & {} \Vert C\Vert _\pi \le \liminf _i \Vert C_{n_i}\Vert _\pi \le \liminf _i \mathcal {N} (u_{n_i})\\&+ {n_i}^{-1} \le \lim _i \mathcal {N} (u_{n_i}) = \liminf \mathcal {N} (u_n), \end{aligned}$$

which concludes the proof. $\square$

This relaxation results suggest to use $$ \mathcal {N} (\cdot )$$ as in Equation (7) as convex texture prior in the continuous setting. There is, however, an issue with that, namely that such a functional cannot be expected to penalize the number of used atoms at all. Indeed, taking some $$C= \sum _{i=1}^l c_i \otimes p_i$$ and assume that $$\Vert C\Vert _\pi = \sum _{i=1}^l \Vert c_i\Vert _\mathcal {M}\Vert p_i\Vert _s$$. Now note that we can split any summand $$c_{i_0} \otimes p_{i_0}$$ as follows: Write $$c_{i_0} =c^1_{i_0} + c^2_{i_0} $$ with disjoint support such that $$\Vert c_{i_0} \Vert _\mathcal {M}= \Vert c^1_{i_0} \Vert _\mathcal {M}+ \Vert c^2_{i_0}\Vert _\mathcal {M}$$. Then, we can rewrite

$$\begin{aligned} c_{i_0} \otimes p_{i_0} = c^1_{i_0}\otimes p_{i_0} + c^2_{i_0}\otimes p_{i_0} \end{aligned}$$

which gives a different representation of *C* by increasing the number of atoms without changing the cost of the projective norm. Hence, in order to maintain the original motivation of the approach to enforce a limited number of atoms, we need to add an additional penalty on *C* for the lifted texture prior.

### Adding a Rank Penalization

Considering the discrete setting and the representation of the tensor *C* as a matrix, the number of used atoms corresponds to the rank of the matrix, for which it is well known that the nuclear norm constitutes a convex relaxation [25]. This construction can in principle also be transferred to general tensor products of Banach spaces via the identification (see Proposition 23 in “Appendix”)

$$\begin{aligned}&C = \sum _{i=1}^\infty x_i \otimes y_i \in X^* \otimes _\pi Y^* \quad \longleftrightarrow \quad T_C \in \mathcal {L}(X,Y^*) \\&\text {where} \quad T_C(x) = \sum _{i=1}^\infty x_i(x)y_i \end{aligned}$$

and the norm

$$\begin{aligned}&\Vert C\Vert _\text {nuc}= \Vert T_C\Vert _\text {nuc}= \inf \left\{ \sum _{i=1}^\infty \sigma _i \, \left| \right. \, T_C (x)\right. \\&\quad \left. = \sum _{i=1}^\infty \sigma _i x_i(x)y_i \text { s.t. } \Vert x_i\Vert _{X^*}\le 1, \Vert y_i\Vert _{Y^*} \le 1 \right\} . \end{aligned}$$

It is important to realize, however, that the nuclear norm of operators depends on the underlying spaces and in fact coincides with the projective norm in the tensor product space (see Proposition 23). Hence, adding the nuclear norm in $X_s$ does not change anything, and more generally, whenever one of the underlying spaces is equipped with an $L^1$-type norm, we cannot expect a rank-penalizing effect (consider the example of the previous section).

On the other hand, going back to the nuclear norm of a matrix in the discrete setting, we see that it relies on orthogonality and an inner product structure and that the underlying norm is the Euclidean inner product norm. Hence, an appropriate generalization of a rank-penalizing nuclear norm needs to be built on a Hilbert space setting. Indeed, it is easy to see that any operator between Banach spaces with a finite nuclear norm is compact, and in particular for any $T \in \mathcal {L}(H_1,H_2)$ with finite nuclear norm and $H_1,H_1$ Hilbert spaces, there are orthonormal systems $(x_i)_i$, $(y_i)_i$ and uniquely defined singular values $(\sigma _i)_i$ such that

$$\begin{aligned} Tx = \sum _{i=1}^\infty \sigma _i (x,x_i)y_i \quad \text {and in addition}\quad \Vert T\Vert _\text {nuc}= \sum _{i=1}^\infty \sigma _i. \end{aligned}$$

Motivated by this, we aim to define an $L^2$-based nuclear norm as extended real-valued function on $X_s$ as convex surrogate of a rank penalization. To this aim, we consider from now on the case $s=2$. Remember that the tensor product $X \otimes Y$ of two spaces *X*, *Y* is defined as the vector space spanned by linear mappings $x \otimes y$ on the space of bilinear forms on $X \times Y$, which are given as $x \otimes y (B) = B(x,y)$. Now since $L^2(\Omega _\Sigma )$ can be regarded as subspace of $\mathcal {M}(\Omega _\Sigma )$, also $L^2(\Omega _\Sigma ) \otimes L^2(\Sigma )$ can be regarded as subspace of $\mathcal {M}(\Omega _\Sigma ) \otimes L^2(\Sigma ).$ Further, defining for $C \in L^2(\Omega _\Sigma ) \otimes L^2(\Sigma )$,

$$\begin{aligned}&\Vert C\Vert _{\pi ,L^2\otimes L^2}:= \inf \left\{ \sum _{i=1}^n \Vert x_i\Vert _{2} \Vert y_i\Vert _{2} \, \left| \right. \, C \right. \\&\quad \left. = \sum _{i=1}^n x_i \otimes y_i,\, x_i \in L^2(\Omega _\Sigma ), \, y_i \in L^2(\Sigma ), \, n \in \mathbb {N}\right\} , \end{aligned}$$

we get that $\Vert \cdot \Vert _\pi \le \Theta \Vert \cdot \Vert _{\pi ,L^2\otimes L^2}$ for a constant $\Theta >0$, and hence, also the completion $L^2(\Omega _\Sigma ) \otimes _\pi L^2(\Sigma )$ can be regarded as subspace of $\mathcal {M}(\Omega _\Sigma ) \otimes _\pi L^2(\Sigma )$. Further, $L^2(\Omega _\Sigma ) \otimes _\pi L^2(\Sigma )$ can be identified with the space of nuclear operators ${\mathcal {N}}(L^2(\Omega _\Sigma ),L^2(\Sigma ))$ as above such that

$$\begin{aligned} \Vert C\Vert _{\pi ,L^2\otimes L^2}= & {} \sum _{i=1}^\infty \sigma _i(T_C) \\&\text {with } (\sigma _i(T_C))_i \text { the singular values of }T_C. \end{aligned}$$

Using this, and introducing a potential function $\phi :[0,\infty ) \rightarrow [0,\infty )$, we define for $C \in X_2$,

8 $$\begin{aligned} \Vert C\Vert _{\text {nuc},\phi } := {\left\{ \begin{array}{ll} \sum _{i=1}^\infty \phi (\sigma _i(T_C)) &{}\text {if } C \in L^2(\Omega _\Sigma ) \otimes _\pi L^2(\Sigma ) ,\\ \infty &{} \text { else.} \end{array}\right. } \end{aligned}$$

We will mostly focus on the case $\phi (x)=x$, in which $\Vert \cdot \Vert _{\text {nuc},\phi }$ coincides with an extension of the nuclear norm and can be interpreted as convex relaxation of the rank. However, since we observed a significant improvement in some cases in practice by choosing $\phi$ to be a semi-convex potential function, i.e., a function such that $\phi + \tau |\cdot |^2$ is convex for $\tau$ sufficiently small, we include the more general situation in the theory.

#### Remark 5

(Sparsity and low-rank) It is important to note that $\Vert C\Vert _{\text {nuc},\phi }< \infty$ restricts *C* to be contained in the smoother space $L^2(\Omega _\Sigma ) \otimes _\pi L^2(\Sigma )$ and in particular does not allow for simple tensors $\sum _{i=1}^k c_i \otimes p_i$ with the $c_i$’s being composed of delta peaks. Thus, we observe some inconsistency of a rank penalization via the nuclear norm and a pointwise sparsity penalty, which is only visible in the continuous setting via regularity of functions. Nevertheless, such an inconsistency has already been observed in the finite-dimensional setting in the context of compressed sensing for low-rank AND sparse matrices, manifested via a poor performance of the sum of a nuclear norm and $\ell ^1$ norm for exact recovery (see [44]). As a result, there exist many studies on improved, convex priors for the recovery of low-rank and sparse matrices, see, for instance, [19, 49, 50]. While such improved priors can be expected to be highly beneficial for our setting, the question does not seem to be solved in such a way that can be readily applied in our setting.

One direct way to circumvent this inconsistency would be to include an additional smoothing operator for *C* as follows: Take $S \in \mathcal {L}(\mathcal {M}(\Omega _\Sigma ),\mathcal {M}(\Omega _\Sigma ))$ such that $\text {range}(S) \subset L^2(\Omega _\Sigma )$ to be a weak*-to-weak* continuous linear operator and define the operator ${\hat{S}}:X_2 \rightarrow X_2$ as ${\hat{S}}:=S \otimes I_{L^2}$, where $I_{L^2}$ denotes the identity in $L^2(\Sigma )$. Then one could alternatively also use $\Vert SC\Vert _{\text {nuc}}$ as alternative for penalizing the rank of *C* while still allowing *C* to be a general measure. Indeed, in the discrete setting, by choosing *S* also to be injective, we even obtain the equality $\text {rank}(SC) = \text {rank}(C)$ (where we interpret *C* and *SC* as matrices). In practice, however, we did not observe an improvement by including such a smoothing and thus do not include ${\hat{S}}$ in our model.

#### Remark 6

(Structured matrix completion) We would also like to highlight the structured-matrix-completion viewpoint on the difficulty of low-rank and sparse recovery. In this context, the work [29] discusses conditions for global optimality of solutions to the non-convex matrix decomposition problem

9 $$\begin{aligned} \min _{U \in \mathbb {R}^{N \times k},V \in \mathbb {R}^{n \times k} } \ell (Y,UV^T) + \sum _{i=1}^k \theta (U_i,V_i) \end{aligned}$$

where $\ell$ measures the loss w.r.t. some given data and $\theta (\cdot ,\cdot )$ allows to enforce structural assumptions on the factors *U*, *V*. For this problem, [29] shows that rank-deficient local solutions are global solutions to a convex minorant obtained allowing *k* to become arbitrary large (formally, choose $k=\infty$) and, consequently, also globally optimal for the original problem. Choosing $\ell (Y,UV^T) = 0$ if $Y = {\hat{K}}(UV^T)$ and infinity else, where ${\hat{K}}$ is a discrete version of the lifted convolution operator, and $\theta (U_i,V_i) = \Vert U_i\Vert _2 \Vert V_i\Vert _1$, we see that (for simplicity ignoring the optional additional atom constraints) the convolutional Lasso prior $\mathcal {N}_\text {cl,s}$ of Equation (5) can be regarded as special case of (9). Viewed in this way, our above results show that the convex minorant obtained with $k=\infty$ is in fact the convex relaxation, i.e., the largest possible convex minorant, of the entire problem including the data- and the convolution term. But again, as discussed in Sect. 3.2, we cannot expect a rank-penalizing effect of the convex relaxation obtained in this way. An alternative, as mentioned in [29], would be to choose $\theta (U_i,V_i) = \Vert U_i\Vert _2^2 + \Vert V_i\Vert _2^2 + \gamma \Vert V_i\Vert _1$. Indeed, while in this situation it is not clear if the convex minorant with $r= \infty$ is the convex relaxation, the former still provides a convex energy from which one would expect a rank-penalizing effect. Thus, this would potentially be an alternative approach that could be used in our context with the advantage of avoiding the lifting but the difficulty of finding rank-deficient local minima of a non-convex energy.

### Well-Posedness and a Cartoon–Texture Model

Including $\Vert C\Vert _{\text {nuc},\phi }$ for $C \in X_2$ as additional penalty in our model, we ultimately arrive at the following variational texture prior in the tensor product space $X_2:=\mathcal {M}(\Omega _\Sigma )\otimes _\pi L^2(\Sigma )$, which is convex whenever $\phi$ is convex, in particular for $\phi (x) = |x|$.

10 $$\begin{aligned} \mathcal {N} _\nu (v)= & {} \inf _ {C \in X_2} \, \nu \Vert C\Vert _{ \pi } + (1-\nu )\Vert C\Vert _{\text {nuc},\phi } \nonumber \\&\quad \text {s.t. } \left\{ \begin{aligned}&{\hat{M}}C = 0 , \\&v = {\hat{K}}C \text { in } \Omega , \end{aligned} \right. \end{aligned}$$

where $\nu \in (0,1)$ is a parameter balancing the sparsity and the rank penalty.

In order to employ $\mathcal {N} _\nu$ as a regularization term in an inverse problems setting, we need to obtain some lower semi-continuity and coercivity properties. As a first step, the following lemma, which is partially inspired by techniques used in [10, Lemma 3.2], shows that, under some weak conditions on $\phi$, $\Vert \cdot \Vert _{\text {nuc},\phi }$ defines a weak* lower semi-continuous function on $X_2$.

#### Lemma 7

Assume that $\phi :[0,\infty ) \rightarrow [0,\infty )$ is lower semi-continuous, non-decreasing, that

- $\phi (x) \rightarrow \infty$ for $x \rightarrow \infty$ and that
- there exist $\epsilon ,\eta >0$ such that $\phi (x) \ge \eta x$ for $0 \le x < \epsilon$.

Then, the functional $\Vert \cdot \Vert _{\text {nuc},\phi }:X_2 \rightarrow {\overline{R}}$ defined as in (8) is lower semi-continuous w.r.t. weak* convergence in $X_2$.

#### Proof

Take $(C_n)_n \subset X_2$ weak* converging to some $C \in X_2$ for which, w.l.o.g., we assume that

$$\begin{aligned} \liminf _n \Vert C_n\Vert _{\text {nuc},\phi } = \lim _n \Vert C_n\Vert _{\text {nuc},\phi }. \end{aligned}$$

We only need to consider the case that $(\Vert C_n\Vert _{\text {nuc},\phi })_n$ is bounded, otherwise the assertion follows trivially. Hence, we can write $T_{C_n}(x) = \sum _{i=1}^\infty \sigma _i^n (x_i^n,x)y_i^n$ such that $\Vert C_n\Vert _{\text {nuc},\phi } = \sum _{i=1}^\infty \phi (\sigma _i^n)$. Now we aim to bound $(\Vert C_n\Vert _{\pi ,L^2 \otimes L^2})_n$ in terms of $(\Vert C_n\Vert _{\text {nuc},\phi })_n$. To this aim, first note that the assumptions in $\phi$ imply that for any $\epsilon ' >0$ there is $\eta '>0$ such that $\phi (x) \ge \eta ' x$ for all $x < \epsilon '$. Also, $\phi (\sigma _i^n) \le \Vert C_n\Vert _{\text {nuc},\phi }$ for any *i*, *n* and via a direct contradiction argument it follows that there exists ${\hat{\epsilon }}>0$ such that $\sigma _i^n < {\hat{\epsilon }}$ for all *i*, *n*. Picking ${\hat{\eta }}$ such that $\phi (x) \ge {\hat{\eta }}x$ for all $x < {\hat{\epsilon }}$, we obtain

$$\begin{aligned} \Vert C_n\Vert _{\text {nuc},\phi } = \sum _{i=1}^\infty \phi (\sigma _i^n ) \ge {\hat{\eta }} \sum _{i=1}^\infty \sigma _i^n = {\hat{\eta }} \Vert C_n\Vert _{\pi ,L^2 \otimes L^2} , \end{aligned}$$

hence $(C_n)_n$ is also bounded as a sequence in $L^2(\Omega _\Sigma ) \otimes _\pi L^2(\Sigma )$ and admits a (non-relabeled) subsequence weak* converging to some ${\hat{C}} \in L^2(\Omega _\Sigma ) \otimes _\pi L^2(\Sigma )$, with $L^2(\Omega _\Sigma ) \otimes _\mathfrak {i}L^2(\Sigma )$ being the predual space. By the inclusion $C_0(\Omega _\Sigma ) \otimes _\mathfrak {i}L^2(\Sigma ) \subset L^2(\Omega _\Sigma ) \otimes _\mathfrak {i}L^2(\Sigma )$ and uniqueness of the weak* limit, we finally get ${\hat{C}}=C \in L^2(\Omega _\Sigma ) \otimes _\mathfrak {i}L^2(\Sigma )$ and can write $T_Cx = \sum _{i=1}^\infty \sigma _i (x_i,x) y_i$ and $\Vert C\Vert _{\text {nuc},\phi } = \sum _{i=1}^\infty \sigma _i$. By lower semi-continuity of $\Vert \cdot \Vert _{\text {nuc}}$, this would suffice to conclude in the case $\phi (x) = x$. For the more general case, we need to show a pointwise lim-inf property of the singular values. To this aim, note that by the Courant–Fischer min–max principle (see, for instance, [12, Problem 37]) for any compact operator $T \in {\mathcal {L}}(H_1,H_2)$ with $H_1,H_2$ Hilbert spaces and $\lambda _k$ the *k*-th singular value of *T* sorted in descending order, we have

$$\begin{aligned} \lambda _k = \sup _{\dim (V)=k} \min _{x \in V, \Vert x\Vert =1} \Vert Tx\Vert _{H_2}. \end{aligned}$$

Now consider $k\in \mathbb {N}$ fixed. For any subspace *V* with $\dim (V)= k$, the minimum in the equation above is achieved, and hence, we can denote $x_V$ to be a minimizer and define $\mathcal {F} _V(T):= \Vert Tx_V\Vert _{H_2}$ such that $\lambda _k = \sup _{\dim (V)=k} \mathcal {F} _V(T)$. Since weak* convergence of a sequence $(T_n)$ to *T* in $L^2(\Omega _\Sigma ) \otimes _\pi L^2(\Sigma )$ implies in particular $T_{n}(x) \rightharpoonup T(x)$ for all *x*, by lower semi-continuity of the norm $\Vert \cdot \Vert _{H_2}$ it follows that $\mathcal {F} _V$ is lower semi-continuous with respect to weak* convergence. Hence, this is also true for the function $T \mapsto \lambda _k(T)$ by being the pointwise supremum of a family of lower semi-continuous functional. Consequently, for the sequence $(T_{C_n})_n$ it follows that $\sigma _k \le \liminf _n \sigma ^n_k$. Finally, by monotonicity and lower semi-continuity of $\phi$ and Fatou’s lemma we conclude

$$\begin{aligned}&\Vert T_{C}\Vert _{\text {nuc},\phi } = \sum _{k} \phi (\sigma _k) \le \sum _{k} \phi (\liminf _n \sigma _k^n)\\&\quad = \sum _{k} \liminf _n \phi (\sigma _k^n) \le \liminf _n \sum _k \phi (\sigma _k^n) \\&\quad \le \liminf _n \Vert T_{C_n}\Vert _{\text {nuc},\phi } . \end{aligned}$$

$$\square $$

The lemma below now establishes the main properties of $\mathcal {N} _\nu$ that in particular allow to employ it as regularization term in an inverse problems setting.

#### Lemma 8

The infimum in the definition of (10) is attained and $\mathcal {N} _\nu :L^q(\Omega ) \rightarrow {\overline{\mathbb {R}}}$ is convex and lower semi-continuous. Further, any sequence $(v_n)_n$ such that $\mathcal {N} _\nu (v_n)$ is bounded admits a subsequence converging weakly in $L^q(\Omega )$.

#### Proof

The proof is quite standard, but we provide it for the readers convenience. Take $$(v_n)_n $$ to be a sequence such that $$( \mathcal {N} _\nu (v_n))_n$$ is bounded. Then, we can pick a sequence $$(C_n)_n$$ in $$X_2$$ such that $${\hat{M}}C_n = 0$$, $$v_n = {\hat{K}}C_n$$ and

$$\begin{aligned} \nu \Vert C_n\Vert _\pi \le \nu \Vert C_n\Vert _\pi + (1-\nu ) \Vert C_n\Vert _{\text {nuc},\phi } \le \mathcal {N} _\nu (v_n) + n^{-1} \end{aligned}$$

This implies that $(C_n)_n$ admits a subsequence $(C_{n_i})_i$ weak* converging to some $C \in X_2$. Now by continuity of ${\hat{M}}$ and ${\hat{K}}$ we have that ${\hat{M}}C = 0$ and that $(v_{n_i})_i = ({\hat{K}}C_{n_i})_i$ is bounded. Hence also $(v_{n_i})_i$ admits a (non-relabeled) subsequence converging weakly to some $v={\hat{K}}C$. This already shows the last assertion. In order to show lower semi-continuity, assume that $(v_n)_n$ converges to some *v* and, without loss of generality, that

$$\begin{aligned} \liminf _n \mathcal {N} _\nu (v_n) = \lim _n \mathcal {N} _\nu (v_n) . \end{aligned}$$

Now this is a particular case of the argumentation above; hence, we can deduce with $(C_n)_n$ as above that

$$\begin{aligned} \mathcal {N} _\nu (v)&\le \nu \Vert C\Vert _\pi + (1-\nu ) \Vert C\Vert _{\text {nuc},\phi } \\&\le \liminf _i \nu \Vert C_{n_i}\Vert _\pi + (1-\nu ) \Vert C_{n_i}\Vert _{\text {nuc},\phi } \\&\le \liminf _i \mathcal {N} _\nu (v_{n_i}) +{n_i}^{-1} = \liminf _n \mathcal {N} _\nu (v_n) \end{aligned}$$

which implies lower semi-continuity. Finally, specializing even more to the case that $$(v_n)_n $$ is the constant sequence $$(v)_n$$, also the claimed existence follows. $$\square $$

In order to model a large class of natural images and to keep the number of atoms needed in the above texture prior low, we combine it with a second part that models cartoon-like images. Doing so, we arrive at the following model

P $$\begin{aligned} \min _{u ,v\in L^q(\Omega )} \lambda \mathcal {D} (Au,f_0) + s_1(\mu ) \mathcal {R} (u- v) + s_2(\mu ) \mathcal {N} _\nu (v) \end{aligned}$$

where we assume $\mathcal {R}$ to be a functional that models cartoon images, $\mathcal {D} (\cdot ,f_0):Y \rightarrow {\overline{\mathbb {R}}}$ is a given data discrepancy, $A \in \mathcal {L}(L^q(\Omega ),Y)$ a forward model and we define the parameter balancing function

11 $$\begin{aligned} s_1(\mu ) = 1 - \min (\mu ,0), \quad s_2(\mu ) = 1 + \max (\mu ,0). \end{aligned}$$

Now we get the following general existence result.

#### Proposition 9

Assume that $\mathcal {R} :L^q(\Omega ) \rightarrow {\overline{R}}$ is convex, lower semi-continuous and that there exists a finite-dimensional subspace $U \subset L^q(\Omega )$ such that for any $u\in L^q(\Omega )$, $v \in U^\perp$, $w \in U$,

$$\begin{aligned} \Vert v \Vert _q \le \Theta \mathcal {R} (v),\quad \text {and}\quad \mathcal {R} (u+w) = \mathcal {R} (u) \end{aligned}$$

with $\Theta >0$ and $U^\perp$ denoting the complement of *U* in $L^q(\Omega )$. Further assume that $A \in \mathcal {L}(L^q(\Omega ),Y)$, $\mathcal {D} (\cdot ,f_0)$ is convex, lower semi-continuous and coercive on the finite-dimensional space *A*(*U*) in the sense that for any two sequences $(u_n^1)_n$, $(u_n^2)_n$ such that $(u_n^1)_n \subset U$, $(u_n^2)_n$ is bounded and $( \mathcal {D} (A(u_n^1 + u_n^2),f_0))_n$ is bounded, also $(\Vert A u^1_n\Vert _q)_n$ is bounded. Then, there exists a solution to (P).

#### Remark 10

Note that, for instance, in case $\mathcal {D}$ satisfies a triangle inequality, the sequence $(u^2_n)$ in the coercivity assumption is not needed, i.e., can be chosen to be zero.

#### Proof

The proof is rather standard, and we provide only a short sketch. Take $((u_n,v_n))_n$ a minimizing sequence for (P). From Lemma 8, we get that $(v_n)_n$ admits a (non-relabeled) weakly convergent subsequence. Now we split $u_n = u_n^1 + u_n^2 \in U + U^\perp$ and $v_n = v_n^1 + v_n^2 \in U + U^\perp$ and by assumption get that $\Vert u^2_n - v^2_n\Vert _q$ is bounded. But since $(\Vert v_n\Vert _q)_n$ is bounded, so is $(\Vert v^2_n\Vert _q)_n$ and consequently also $(\Vert u^2_n\Vert _q)_n$. Now we split again $u_n^1 = u_n^{1,1} + u_n^{1,2} \in \ker (A) \cap U + (\ker (A) \cap U)^\perp$, where the latter denotes the complement of $(\mathrm{ker}(A)\cap U)^\perp \hbox { in } U$, and note that also $(u_n^{1,2} + u_n^2,v_n)$ is a minimizing sequence for (P). Hence, it remains to show that $(u_n^{1,2})_n$ is bounded in order to get a bounded minimizing sequence. To this aim, we note that $(u_n^{1,2})_n \subset (\ker (A) \cap U)^\perp \cap U$ and that *A* is injective on this finite-dimensional space. Hence, $\Vert u_n^{1,2}\Vert _q \le {\tilde{\Theta }} \Vert Au_n^{1,2}\Vert _q$ for some ${\tilde{\Theta }}>0$, and by the coercivity assumption on the data term we finally get that $(\Vert u_n^{1,2}\Vert _q)_n$ is bounded. Hence, also $(u_n^{1,2} + u_n^2)_n$ admits a weakly convergent subsequence in $L^q(\Omega )$ and by continuity of *A* as well as lower semi-continuity of all involved functionals existence of a solution follows. $\square$

#### Remark 11

(Choice of regularization) A particular choice of regularization for $\mathcal {R}$ in (P) that we consider in this paper is $\mathcal {R} = {{\,\mathrm{TGV}\,}}_\alpha ^2$, with ${{\,\mathrm{TGV}\,}}_\alpha ^2$ the second-order total generalized variation functional [9], $q \le d/(d-1)$ and

$$\begin{aligned} Mp:= \left( \int _\Sigma p(x) \,\mathrm {d}x,\int _\Sigma p(x_1,x_2) x_1 \,\mathrm {d}x,\int _\Sigma p(x_1,x_2) x_2 \,\mathrm {d}x \right) . \end{aligned}$$

Since in this case [7, 11]

$$\begin{aligned} \Vert u\Vert _q \le \Theta {{\,\mathrm{TGV}\,}}_\alpha ^2(u) \end{aligned}$$

with $\Theta >0$ and for all $u \in {\mathcal {P}}_1(\Omega )^\perp$, the complement of the first-order polynomials, and ${{\,\mathrm{TGV}\,}}_\alpha ^2$ is invariant on first-order polynomials, the result of Proposition 9 applies.

#### Remark 12

(Norm-type data terms) We also note that the result of Proposition 9 in particular applies to $\mathcal {D} (w,f_0):= \frac{1}{r}\Vert w-f_0\Vert _r ^r$ for any $r \in [1,\infty )$ or $\mathcal {D} (w,f_0):= \Vert w-f\Vert _\infty$, where we extend the norms by infinity to $L^q(\Omega )$ whenever necessary. Indeed, lower semi-continuity of these norms is immediate for both $r\le q$ and $r>q$, and since the coercivity is only required on a finite-dimensional space, it also holds by equivalence of norms.

#### Remark 13

(Inpainting) At last we also remark that the assumptions of Proposition 9 also hold for an inpainting data term defined as

$$\begin{aligned} \mathcal {D} (w,f_0):= {\left\{ \begin{array}{ll} 0 &{}\text {if } w=f_0 \text { a.e. on } \omega \subset \Omega \\ \infty &{} \text {else,} \end{array}\right. } \end{aligned}$$

whenever $\omega$ has non-empty interior. Indeed, lower semi-continuity follows from the fact that $L^q$ convergent sequences admit pointwise convergent subsequences and the coercivity follows from finite dimensionality of *U* and the fact that $\omega$ has non-empty interior.

#### Remark 14

(Regularization in a general setting) We also note that Lemma 8 provides the basis for employing either $N_\nu$ directly or its infimal convolution with a suitable cartoon prior as in Proposition 9 for the regularization of general (potentially nonlinear) inverse problems and with multiple data fidelities, see, for instance, [31, 32] for general results in that direction.

## The Model in a Discrete Setting

This section deals with the discretization of the proposed model and its numerical solution. For the sake of brevity, we provide only the main steps and refer to the publicly available source code [16] for all details.

We define $U = \mathbb {R}^{N \times M}$ to be the space of discrete grayscale images, $W = \mathbb {R}^{(N+n-1) \times (M+n-1)}$ to be the space of coefficient images and $Z = \mathbb {R}^{n \times n}$ to be the space of image atoms for which we assume $n<\min \{N,M\}$ and, for simplicity, only consider a square domain for the atoms. The tensor product of a coefficient image $c\in W$ and a atom $p \in Z$ is given as $(c \otimes p )_{i,j,r,s} = c_{i,j}p_{r,s}$ and the lifted tensor space is given as the four-dimensional space $X = \mathbb {R}^{(N+n-1) \times (M+n-1) \times n \times n}$.

*Texture Norm* The forward operator *K* being the lifting of the convolution $c *p$ and mapping lifted matrices to the vectorized image space is then given as

$$\begin{aligned} (KC)_{i,j} = \sum _{r,s=1}^{n,n} C_{i+n-r,j+n-s,r,s} \end{aligned}$$

and we refer Fig. 1 for a visualization in the one-dimensional case. Note that by extending the first two dimensions of the tensor space to $N+n-1$, $M+n-1$ we allow to place an atom at any position where it still effects the image, also partially outside the image boundary.

Also we note that, in order to reduce dimensionality and accelerate the computation, we introduce a stride parameter $\eta \in \mathbb {N}$ in practice which introduces a stride on the possible atom positions. That is, the lifted tensor space and forward operator are reduced in such a way that the grid of possible atom positions in the image is essentially $\{ (\eta i,\eta j) \, \left| \right. \, i,j\in \mathbb {N}, (\eta i,\eta j) \in \{1,\ldots ,N\} \times \{1,\ldots ,M\} \}$. This reduces the dimension of the tensor space by a factor $\eta ^{-2}$, while for $\eta >1$ it naturally does not allow for arbitrary atom positions anymore and for $\eta =n$ it corresponds to only allowing non-overlapping atoms. In order to allow for atoms being placed next to each other, it is important to choose $\eta$ to be a divisor of the atom-domain size *n* and we used $n=15$ and $\eta =3$ in all experiments of the paper. In order to avoid extensive indexing and case distinctions, however, we only consider the case $\eta =1$ here and refer to the source code [16] for the general case.

A straightforward computation shows that, in the discrete lifted tensor space, the projective norm corresponding to discrete $\Vert \cdot \Vert _1$ and $\Vert \cdot \Vert _2$ norms for the coefficient images and atoms, respectively, is given as a mixed 1-2 norm as

$$\begin{aligned} \Vert C\Vert _\pi = \Vert C\Vert _{1,2} = \sum _{i,j=1}^{N,M} \sqrt{ \sum _{r,s=1}^{n,n} C_{i,j,r,s}^2} . \end{aligned}$$

**Fig. 3:**
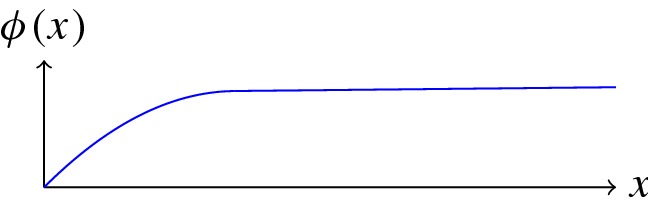
Visualization of the potential $\phi$.

The nuclear norm for a potential $\phi$ on the other hand reduces to the evaluation of $\phi$ on the singular values of a matrix reshaping of the lifted tensors and is given as

$$\begin{aligned}&\Vert C\Vert _{\text {nuc},\phi } = \sum _{i=1}^{nn} \phi (\sigma _i), \\&\quad \text {with }(\sigma _i)_i\text { the singular values of }B = [C_{(NM,nn)}]. \end{aligned}$$

where $[C_{(NM,nn)}]$ denotes a reshaping of the tensor *C* to a matrix of dimensions $NM \times nn$. For the potential function $\phi$, we consider two choices: Mostly we are interested in $\phi (x) = x$ which yields a convex texture model and enforces sparsity of the singular values. A second choice we consider is $\phi :[0,\infty ) \rightarrow [0,\infty )$ given as

12 $$\begin{aligned} \phi (x) = {\left\{ \begin{array}{ll} x - \epsilon \delta x^2 &{} x \in [0,\frac{1}{2\epsilon }] \\ (1-\delta ) x + \frac{\delta }{4 \epsilon } &{} \text {else,}\end{array}\right. } \end{aligned}$$

where $\delta < 1$, $\delta \approx 1$ and $\epsilon >0$, see Fig. 3. It is easy to see that $\phi$ fulfills the assumptions of Lemma 7 and that $\phi$ is semi-convex, i.e., $\phi + \rho |\cdot |^2$ is convex for $\rho >\delta \epsilon$. While the results of Sect. 3 hold for this setting even without the semi-convexity assumption, we cannot in general expect to obtain an algorithm that provably delivers a globally optimal solution in the semi-convex (or generally non-convex) case. The reason for using a semi-convex potential rather than a arbitrary non-convex one is twofold: First, for a suitably small stepsize $\tau$ the proximal mapping

$$\begin{aligned} {{\,\mathrm{prox}\,}}_{\tau ,\phi }({\hat{u}}) = \mathop {\hbox {argmin}}\limits _u \frac{\Vert u-{\hat{u}}\Vert _2 ^2}{2\tau } + \phi (u) \end{aligned}$$

is well defined and hence proximal-point-type algorithms are applicable at least conceptually. Second, since we employ $\phi$ on the singular values of the lifted matrices *C*, it will be important for numerical feasibility of the algorithm that the corresponding proximal mapping on *C* can be reduced to a proximal mapping on the singular values. While this is not obvious for a general choice of $\phi$, it is true (see Lemma 15) for semi-convex $\phi$ with suitable parameter choices.

*Cartoon Prior* As cartoon prior we employ the second-order total generalized variation functional which we define for fixed parameters $(\alpha _0,\alpha _1) = (\sqrt{2},1)$ and a discrete image $u \in U$ as

$$\begin{aligned} {{\,\mathrm{TGV}\,}}_\alpha ^2(u) = \min _{v \in U^2} \alpha _1 \Vert \nabla u - v \Vert _1 + \alpha _0 \Vert \mathcal {E}v\Vert _1. \end{aligned}$$

Here, $\nabla$ and $\mathcal {E}$ denote discretized gradient and symmetrized Jacobian operators, respectively, and we refer to [8] and the source code [16] for details on a discretization of ${{\,\mathrm{TGV}\,}}_\alpha ^2$. To ensure a certain orthogonality of the cartoon and texture parts, we further define the operator *M* that incorporates atom constraints, to evaluate the 0th and 1st moments of the atoms, which in the lifted setting yields

$$\begin{aligned} (MC)_{i,j}:= \left( \sum _{r,s=1}^{n,n} C_{i,j,r,s},\sum _{r,s=1}^{n,n} r C_{i,j,r,s}, \sum _{r,s=1}^{n,n} s C_{i,j,r,s}\right) . \end{aligned}$$

The discrete version of (P) is then given as

DP $$\begin{aligned}&\min _{ \begin{array}{c} u\in U ,C \in X \\ MC = 0 \end{array}} \lambda \mathcal {D} (Au,f_0) + s_1(\mu ) {{\,\mathrm{TGV}\,}}_\alpha ^2(u-KC) \\&\quad +\,s_2(\mu )\left( \nu \Vert C\Vert _{1,2} + (1-\nu )\Vert C\Vert _{\text {nuc},\phi } \right) , \end{aligned}$$

where the parameter balancing functions $s_1,s_2$ are given as in (11) and the model depends on three parameters $\lambda ,\mu ,\nu$, with $\lambda$ defining the trade-off between data and regularization, $\mu$ defining the trade-off between the cartoon and the texture parts and $\nu$ defining the trade-off between sparsity and low rank of the tensor *C*.

*Numerical Solution* For the numerical solution of (DP), we employ the primal–dual algorithm of [17]. Since the concrete form of the algorithm depends on whether the proximal mapping of the data term $u \mapsto \mathcal {D} (Au,f_0)$ is explicit or not, in order to allow for a unified version as in Algorithm 1, we replace the data term $\mathcal {D} (Au,f_0)$ by

$$\begin{aligned} \mathcal {D} _1(Au,f_0) + \mathcal {D} _2(u,f_0) \end{aligned}$$

where we assume the proximal mappings of $v \mapsto \mathcal {D} _i(v,f_0)$ to be explicit and, depending on the concrete application, set either $\mathcal {D} _1$ or $\mathcal {D} _2$ to be the constant zero function.

Denoting by $g^*(v):= \sup _{w} (v,w) -g(w)$ the convex conjugate of a function *g*, with $(\cdot ,\cdot )$ being the standard inner product of the sum of all pointwise products of entries of *v* and *w*, we reformulate (DP) to a saddle-point problem asHere, the dual variables $(p,q,d,r,m) \in (U^2,U^3,A(U),U,U^3)$ are in the image space of the corresponding operators, $\mathcal {I}_S(z) = 0$ if $z \in S$ and $\mathcal {I}_S(z) = \infty$ else, $\{ \Vert \cdot \Vert _\infty \le \delta \}:= \{ z \, \left| \right. \, \Vert z\Vert _\infty \le \delta \}$ with $\Vert z \Vert _\infty = \Vert (z_1,\ldots ,z_l) \Vert _\infty = \sup _{i,j} \sqrt{\sum _{s=1}^l (z_{i,j}^s)^2}$ a pointwise infinity norm on $z \in U^l$. The operator *E* and the functional $\mathcal {G}$ are given as

$$\begin{aligned} E(u,v,C)= & {} (\nabla u - \nabla KC - v,\mathcal {E}v,Au,C,MC), \\&\mathcal {G} (x) = \mathcal {G} (u,v,C) = \lambda \mathcal {D} _2(u,f_0)\\&+ s_2(\mu ) (1-\nu ) \Vert C\Vert _{\text {nuc},\phi } \end{aligned}$$

and $\mathcal {F} ^*(y) = \mathcal {F} ^*(p,q,d,r,m)$ summarizes all the conjugate functionals as above. Applying the algorithm of [17] to this reformulation yields the numerical scheme as in Algorithm 1.
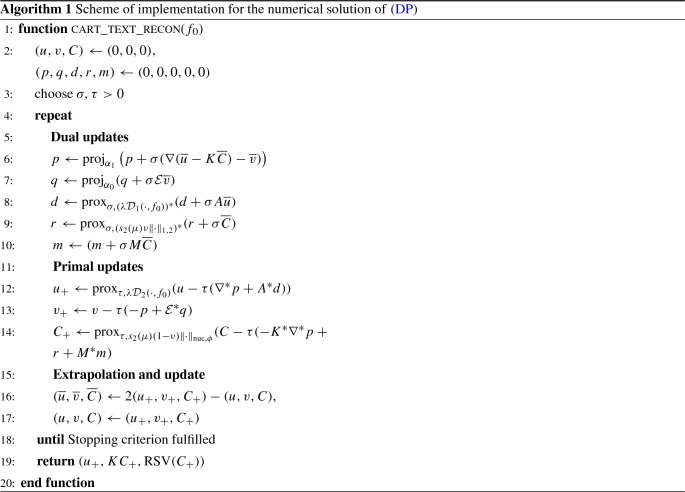


Note that, we set either $\mathcal {D} _1(\cdot ,f_0) \equiv 0$ such that the dual variable *d* is constant 0 and line 9 of the algorithm can be skipped, or we set $\mathcal {D} _2(\cdot ,f_0) \equiv 0$ such that the proximal mapping in line 13 reduces to the identity. The concrete choice of $\mathcal {D} _1,\, \mathcal {D} _2$ and the proximal mappings will be given in the corresponding experimental sections. All other proximal mappings can be computed explicitly and reasonably fast: The mappings ${{\,\mathrm{proj}\,}}_{\alpha _1}$ and ${{\,\mathrm{proj}\,}}_{\alpha _1}$ can be computed as pointwise projections to the $L^\infty$-ball (see, for instance, [8]) and the mapping ${{\,\mathrm{prox}\,}}_{\sigma ,(s_2(\mu )\nu \Vert \cdot \Vert _{1,2})^*}$ is a similar projection given as

$$\begin{aligned}&{{\,\mathrm{prox}\,}}_{\sigma ,(s_2(\mu )\nu \Vert \cdot \Vert _{1,2})^*}(C)_{i,j,l,s} \\&\quad = C_{i,j,l,s}/ \left( \max \left\{ 1,(\sum _{l,s=1}^{n,n} C_{i,j,l,s}^2)^{1/2}/ (s_2(\mu )\nu ) \right\} \right) . \end{aligned}$$

Most of the computational effort lies in the computation of ${{\,\mathrm{prox}\,}}_{\tau ,s_2(\mu )(1-\nu )\Vert \cdot \Vert _{\text {nuc},\phi }}$, which, as the following lemma shows, can be computed via an SVD and a proximal mapping on the singular values.

### Lemma 15

Let $\phi :[0,\infty ) \rightarrow [0,\infty )$ be a differentiable and increasing function and $\tau ,\rho >0$ be such that $x \mapsto \frac{x^2}{2\tau }+ \rho \phi (x)$ is convex on $[0,\infty )$. Then, the proximal mapping of $\rho \Vert \cdot \Vert _{\text {nuc},\phi }$ for parameter $\tau$ is given as

$$\begin{aligned} {{\,\mathrm{prox}\,}}_{\tau ,\rho \Vert \cdot \Vert _{\text {nuc},\phi }} (C) = [ ( U{{\,\mathrm{diag}\,}}(({{\,\mathrm{prox}\,}}_{\tau ,\rho \phi }(\sigma _i))_i)V^T)_{((N,M,n,n))}] \end{aligned}$$

where $[C_{(NM,nn)}] = U \Sigma V^T$ is the SVD of $[C_{(NM,nn)}]$ and for $x_0 \ge 0$

$$\begin{aligned} {{\,\mathrm{prox}\,}}_{\tau ,\rho \phi }(x_0) = \min _{x} \frac{|x-x_0|^2}{2\tau } + \rho \phi (|x|) . \end{aligned}$$

In particular, in case $\phi (x) = x$ we have

$$\begin{aligned} {{\,\mathrm{prox}\,}}_{\tau ,\rho \phi }(x_0) = {\left\{ \begin{array}{ll} 0 &{} \text {if } \quad 0 \le x_0 \le \tau \rho , \\ x_0 - \tau \rho &{} \text {else,}\\ \end{array}\right. } \end{aligned}$$

and in case

$$\begin{aligned} \phi (x) = {\left\{ \begin{array}{ll} x - \epsilon \delta x^2 &{}\text {if } x \in [0,\frac{1}{2\epsilon }], \\ (1-\delta ) x + \frac{\delta }{4 \epsilon } &{} \text {else,} \end{array}\right. } \end{aligned}$$

we have that $x \mapsto \frac{x^2}{2\tau }+ \rho \phi (x)$ is convex whenever $\tau \le \frac{1}{2\epsilon \delta \rho }$ and in this case

$$\begin{aligned} {{\,\mathrm{prox}\,}}_{\tau ,\rho \phi }(x_0) = {\left\{ \begin{array}{ll} 0 &{} \text {if } \quad 0 \le x_0 \le \tau \rho ,\\ \frac{x_0 - \tau \rho }{1 - 2 \epsilon \delta \tau \rho } &{}\text {if } \quad \tau \rho< x_0 \le \frac{1}{2 \epsilon } + \tau \rho (1-\delta ), \\ x_0 - \tau \rho (1-\delta ) &{}\text {if } \quad \frac{1}{2 \epsilon } + \tau \rho (1-\delta ) < x_0 .\\ \end{array}\right. } \end{aligned}$$

### Proof

At first note that it suffices to consider $\rho \Vert \cdot \Vert _{\text {nuc},\phi }$ as a function on matrices and show the assertion without the reshaping operation. For any matrix *B*, we denote by $B = U_B \Sigma _B V^T _B$ the SVD of *B* and $\Sigma _B = \text {diag}((\sigma ^B_i)_i)$ contains the singular values sorted in non-increasing order, where $\Sigma _B$ is uniquely determined by *B* and $U_B,V_B$ are chosen to be suitable orthonormal matrices.

We first show that $G(B):= \frac{\Vert B\Vert ^2_2}{2 \tau } + \rho \Vert B \Vert _{\text {nuc},\phi }$ is convex. For $\lambda \in [0,1]$, $B_1,B_2$ matrices, we get by subadditivity of the singular values (see, for instance, [60]) that

$$\begin{aligned}&G(\lambda B_1 + (1-\lambda ) B_2)\\&= \sum _{i} \frac{1}{2\tau }(\sigma _i^{\lambda B_1 + (1-\lambda ) B_2 } )^2 + \rho \phi (\sigma _i^{\lambda B_1 + (1-\lambda ) B_2}) \\&\le \sum _{i} \frac{1}{2\tau }(\lambda \sigma ^{B_1}_i + (1-\lambda ) \sigma ^{B_2}_i )^2 + \rho \phi (\lambda \sigma ^{B_1}_i + (1-\lambda ) \sigma ^{B_2}_i)\\&\le \sum _{i} \frac{\lambda }{2\tau } (\sigma ^{B_1}_i )^2 + \frac{1-\lambda }{2\tau }(\sigma ^{B_2}_i)^2 + \rho \lambda \phi (\sigma ^{B_1}_i) + \rho (1-\lambda ) \phi (\sigma ^{B_2}_i)\\&\le \lambda G({B_1}) + (1-\lambda ) G({B_1}). \end{aligned}$$

Now with $\mathcal {H} (B):=\frac{\Vert B-B_0\Vert ^2_2}{2 \tau } + \rho \Vert B \Vert _{\text {nuc},\phi }$ we get that $\mathcal {H} (B) = G(B) - \frac{1}{2\tau }(2(B,B_0) + \Vert B_0\Vert _2^2)$, and thus, also $\mathcal {H}$ is convex. Hence, first-order optimality conditions are necessary and sufficient and we get (using the derivative of the singular values as in [45]) with $D \mathcal {H}$ the derivative of $\mathcal {H}$ that $B = {{\,\mathrm{prox}\,}}_{\tau ,\rho \Vert \cdot \Vert _{\text {nuc},\phi }} (B_0)$ is equivalent to

$$\begin{aligned} 0&= D \mathcal {H} (B) = (B-B_0) + \tau \rho U_B {{\,\mathrm{diag}\,}}( (\phi ^\prime ( \sigma ^B_i))_i) V_B^T \\&= -B_0 + U_B ( \Sigma _B + \tau \rho {{\,\mathrm{diag}\,}}( (\phi ^\prime ( \sigma ^B_i))_i) )V_B^T \end{aligned}$$

and consequently to

$$\begin{aligned} \sigma ^{B_0}_i = \sigma ^B_i + \tau \rho \phi ^\prime (\sigma ^B_i) \end{aligned}$$

which is equivalent to

$$\begin{aligned} \sigma ^ {B_i} = {{\,\mathrm{prox}\,}}_{\tau ,\rho \phi } (\sigma _i^{B_0}) \end{aligned}$$

as claimed. The other results follow by direct computation. $\square$

Note also that, in Algorithm 1, $KC_+$ returns the part of the image that is represented by the atoms (the “texture part”) and $\text {RSV}(C_+)$ stand for right-singular values of $[(C_+)_{(NM,nn)}]$ and returns the image atoms. For the sake of simplicity, we use a rather high, fixed number of iterations in all experiment but note that, alternatively, a duality-gap-based stopping criterion (see, for instance, [8]) could be used.

**Fig. 4:**
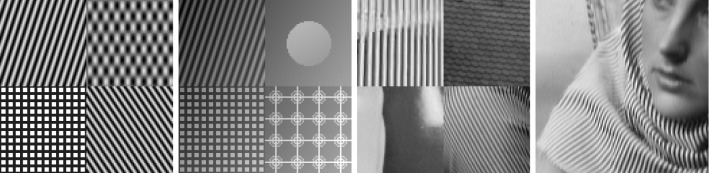
Different test images we will refer to as: Texture, Patches, Mix, Barbara

## Numerical Results

In this section, we present numerical results obtained with the proposed method as well as its variants and compare to existing methods. We will mostly focus on the setting of (DP), where $\phi (x) = |x|$, and we use different data terms $\mathcal {D}$. Hence, the regularization term is convex and consists of ${{\,\mathrm{TGV}\,}}_\alpha ^2$ for the cartoon part and a weighted sum of a nuclear norm and $\ell ^{1,2}$ norm for the texture part. Besides this choice of regularization (called CT-cvx), we will compare to pure ${{\,\mathrm{TGV}\,}}_\alpha ^2$ regularization (called TGV), the setting of (DP) with the semi-convex potential $\phi$ as in (12) (call CT-scvx) and the setting of (DP) with ${{\,\mathrm{TGV}\,}}$ replaced by $\mathcal {I}_{\{0\}}$, i.e., only the texture norm is used for regularization, and $\phi (x) = |x|$ (called TXT). Further, in the last subsection, we also compare to other methods as specified there. For CT-cvx and CT-scvx, we use the algorithm described in the previous section (where convergence can only be ensured for CT-cvx), and for the other variants we use an adaption of the algorithm to the respective special case.

We fix the size of the atom domain to $15 \times 15$ pixel and the stride to 3 pixel (see Sect. 4) for all experiments and use four different test images (see Fig. 4): The first two are synthetic images of size $120\times 120$, containing four different blocks of size $60\times 60$, whose size is a multiple of the chosen atom-domain size. The third and fourth images have size $128 \times 128$ (not being a multiple of the atom-domain size), and the third image contains four sections of real images of size $64 \times 64$ each (again not a multiple of the atom-domain size). All but the first image contain a mixture of texture and cartoon parts. The first four subsections consider only convex variants of our method ($\phi (x) = |x|$), and the last one considers the improvement obtained with a non-convex potential $\phi$ and also compares to other approaches.

Regarding the choice of parameters for all methods, we generally aimed to reduce the number of varying parameters for each method as much as possible such that for each method and type of experiment, at most two parameters need to be optimized. Whenever we incorporate the second-order TGV functional for the cartoon part, we fix the parameters $(\alpha _0,\alpha _1)$ to $(\sqrt{2},1)$. The method CT-cvx then essentially depends on the three parameters $\lambda , \mu ,\nu$. We experienced that the choice of $\nu$ is rather independent of the data and type of experiments; hence, we leave it fixed for all experiments with incomplete or corrupted data, leaving our method with two parameters to be adapted: $\lambda$ defining the trade-off between data and regularization and $\mu$ defining the trade-off between cartoon and texture regularization. For the semi-convex potential, we choose $\nu$ as with the convex one, fix $\delta =0.99$ and use two different choices of $\epsilon$, depending on the type of experiment, hence again leaving two parameters to be adapted. A summary of the parameter choice for all methods is provided in Table 2.

We also note that, whenever we tested a range of different parameters for any method presented below, we show the visually best results in the figure. Those are generally not the ones delivering the best result in terms of peak-signal-to-noise ratio, and for the sake of completeness we also provide in Table 1 the best PSNR result obtained with each method and each experiment over the range of tested parameters.

**Table 1:** Best PSNR result achieved with each method for the parameter test range as specified in Table 2

	Texture	Patches	Mix	Barbara
Inpainting				
TGV	10.32	19.28	20.19	20.58
TXT/ CT-cvx	**17.59**	25.55	**23.38**	23.48
CT-scvx		**32.74**		**23.6**
Denoising				
TGV	11.83	23.96	23.74	23.99
TXT/ CT-cvx	**16.06**	25.91	**26.07**	25.0
CT-scvx		29.4		25.56
CL		29.09		25.14
BM3D		**30.82**		**28.15**
CDL		27.96		25.24
Deconvolution				
TGV			23.72	23.14
CT-cvx			**24.52**	**23.34**

The best result for each experiment is written in bold

### Image-Atom-Learning and Texture Separation

**Fig. 5:**
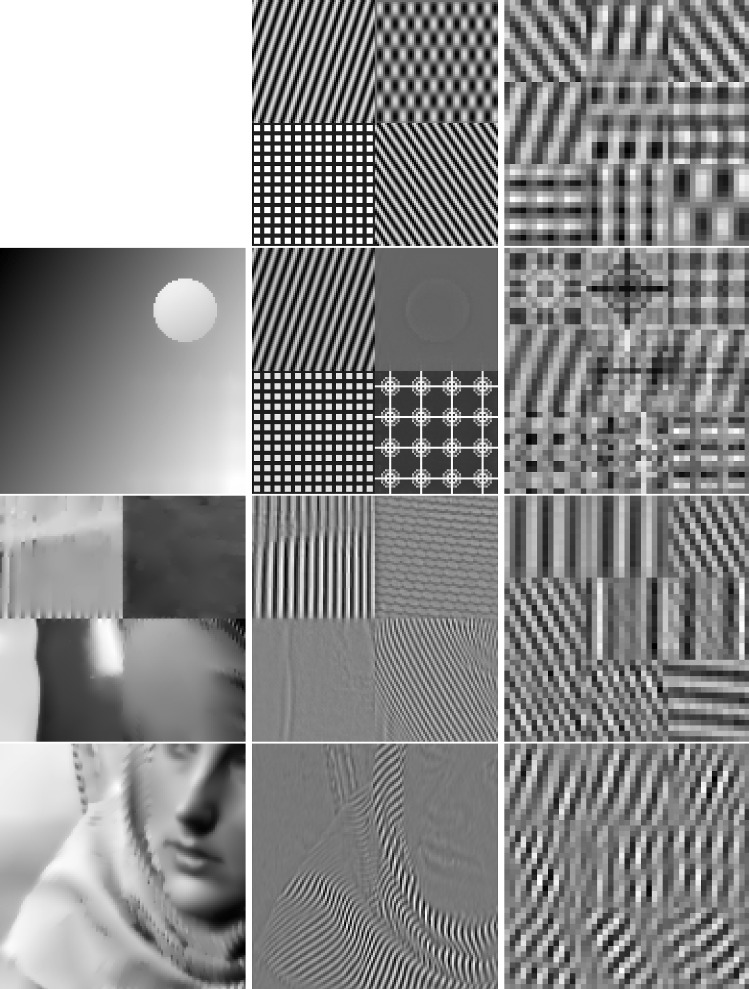
Cartoon–texture decomposition (rows 2–4) and nine most important learned atoms for different test images and the methods TXT (row 1) and CT-cvx (rows 2–4)

As first experiment, we test the method CT-cvx for learning image atoms and texture separation directly on the ground truth images. To this aim, we use

$$\begin{aligned} \mathcal {D} _1 \equiv 0, \qquad \mathcal {D} _2(u,f_0) = \mathcal {I}_{ \{ 0\} } (u-f_0), \end{aligned}$$

and the proximal mapping of $\mathcal {D} _2$ is a simple projection to $f_0$. The results can be found in Fig. 5, where for the pure texture image we used only the texture norm (i.e., the method TXT) without the TGV part for regularization.

It can be observed that the proposed method achieves a good decomposition of cartoon and texture and also is able to learn the most important image structure effectively. While there are some repetitions of shifted structures in the atoms, the different structures are rather well-separated and the first nine atoms corresponding to the nine largest singular values still contain the most important features of the texture parts.

### Inpainting and Learning from Incomplete Data

This section deals with the task of inpainting a partially available image and learning image atoms from these incomplete data. For reference, we also provide results with pure ${{\,\mathrm{TGV}\,}}_\alpha ^2$ regularization (the method TGV). The data fidelity in this case is

$$\begin{aligned} \mathcal {D} _1 \equiv 0, \qquad \mathcal {D} _2(u,f_0) = \mathcal {I}_{\{v \, \left| \right. \, v_{i,j} = (f_0)_{i,j} \text { for } (i,j) \in E\}} (u), \end{aligned}$$

with *E* the index set of known pixels and the proximal mapping of $\mathcal {D} _2$ is a projection to $f_0$ on all points in *E*. Again we use only the texture norm for the first image (the method TXT) and the cartoon–texture functional for the others.

**Fig. 6:**
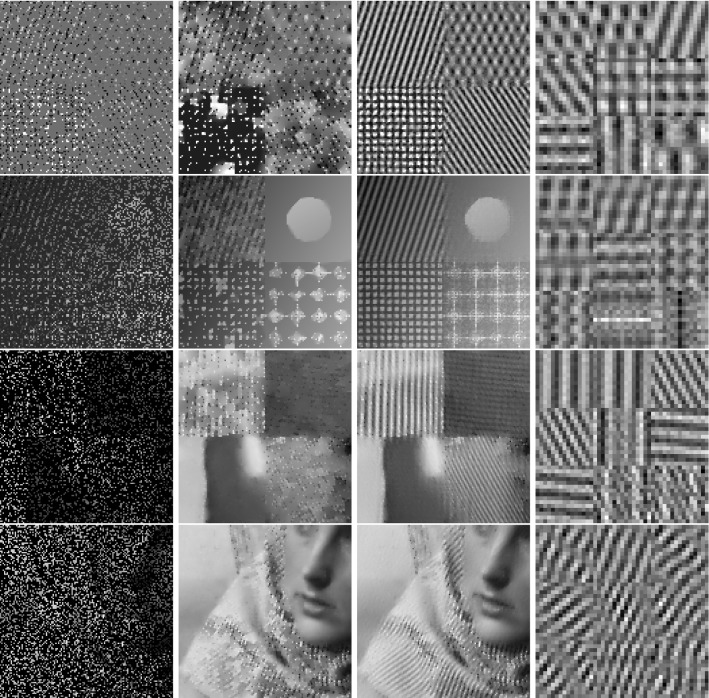
Image inpainting from incomplete data. From left to right: Data, TGV-based reconstruction, proposed method (only TXT in first row), nine most important learned atoms. Rows 1, 3: 20% of pixels, rows 2, 4: 30% of pixels

The results can be found in Fig. 6. For the first and third images, $20\%$ of the pixels were given, while for the other two, $30\%$ were given. It can be seen that our method is generally still able to identify the underlying pattern of the texture part and to reconstruct it reasonably well. Also the learned atoms are reasonable and are in accordance with the ones learned from the full data as in the previous section. In contrast to that, pure ${{\,\mathrm{TGV}\,}}$ regularization (which assumes piecewise smoothness) has no chance to reconstruct the texture patterns. For the cartoon part, both methods are comparable. It can also be observed that the target-like structure in the bottom right of the second image is not reconstructed well and also not well identified with the atoms (only the eighth one contains parts of this structure). The reason might be that due to the size of the repeating structure there is not enough redundant information available to reconstruct it from the missing data. Concerning the optimal PSNR values of Table 1, we can observe a rather strong improvement with CT-cvx compared to TGV.

### Learning and Separation Under Noise

In this section, we test our method for image-atom-learning and denoising with data corrupted by Gaussian noise (with standard deviation 0.5 and 0.1 times the image range for the Texture and the other images, respectively). Again we compare to TGV regularization in this section (but also to other methods in Sect. 5.5) and use the texture norm for the first image (the method TXT). The data fidelity in this case is

$$\begin{aligned} \mathcal {D} _1 \equiv 0, \qquad \mathcal {D} _2(u,f_0) = \frac{1}{2}\Vert u-f_0\Vert _2^2 \end{aligned}$$

and $${{\,\mathrm{prox}\,}}_{\tau ,\lambda \mathcal {D} _2(\cdot ,f_0)}(u) = (u + \tau \lambda f_0)/(1+ \tau \lambda )$$.

**Fig. 7:**
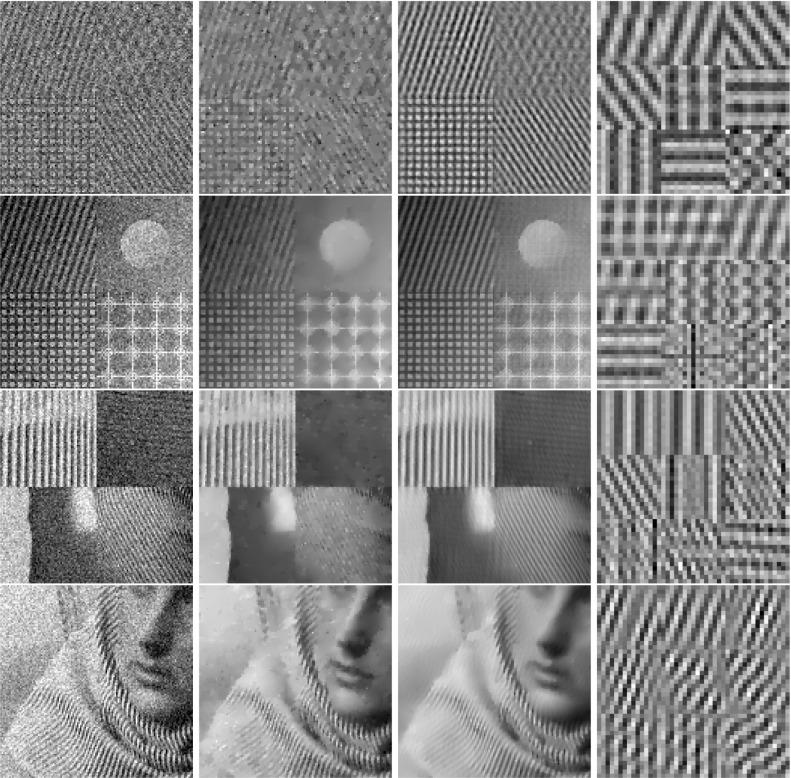
Denoising and atom learning. From left to right: Noisy data, TGV-based reconstruction, proposed method (only TXT for the first image), nine most important learned atoms

The results are provided in Fig. 7. It can be observed that also under the presence of rather strong noise, our method is able to learn some of the main features of the image within the learned atoms. Also the quality of the reconstructed image is improved compared to TGV, in particular for the right-hand side of the *Mix* image, where the top left structure is only visible in the result obtained with CT-cvx. On the other hand, the circle of the *Patches* image obtained with CT-cvx contains some artifacts of the texture part. Regarding the optimal PSNR values of Table 1, the improvement with CT-cvx compared to TGV is still rather significant.

### Deconvolution

This section deals with the learning of image features and image reconstruction in an inverse problem setting, where the forward operator is given as a convolution with a Gaussian kernel (standard deviation 0.25, kernel size $9\times 9$ pixels), and the data are degraded by Gaussian noise with standard deviation 0.025 times the image range. The data fidelity in this case is

$$\begin{aligned} \mathcal {D} _1(u,f_0)= \frac{1}{2}\Vert Au-f_0\Vert _2^2, \qquad \mathcal {D} _2\equiv 0, \end{aligned}$$

with *A* being the convolution operator, and ${{\,\mathrm{prox}\,}}_{\sigma ,(\lambda \mathcal {D} _1(\cdot ,f_0))^*}(u) = (u - \sigma f_0)/(1+\sigma /\lambda )$.

**Fig. 8:**
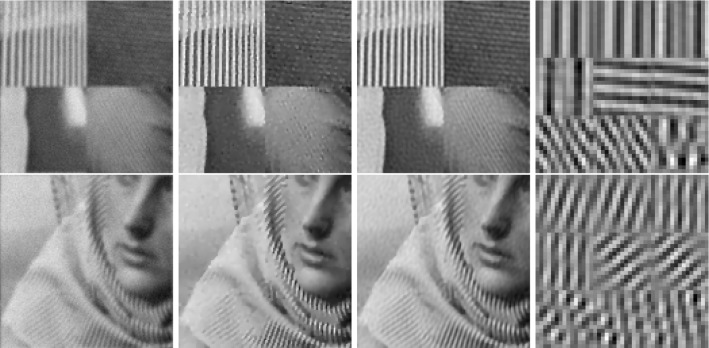
Reconstruction from blurry and noisy data. From left to right: Data, TGV, proposed, learned atoms

We show results for the *Mix* and the *Barbara* image and compare to TGV in Fig. 8. It can be seen that the improvement is comparable to the denoising case. In particular, the method is still able to learn reasonable atoms from the given, blurry data and in particular for the texture parts the improvement is quite significant.

**Fig. 9:**
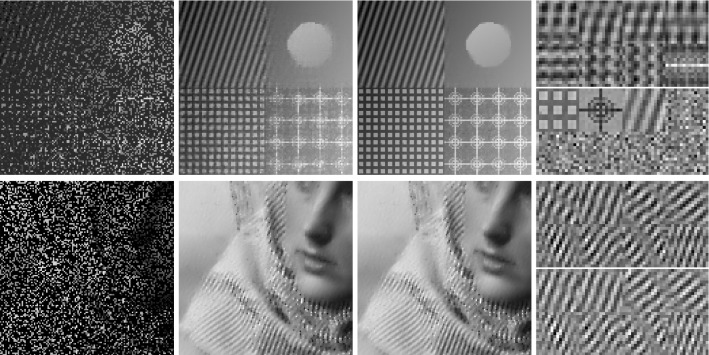
Comparison of CT-cvx and CT-scvx for inpainting with 30% of the pixels given. From left to right: Data, convex, semi-convex, convex atoms (top), semi-convex atoms (bottom)

### Comparison

This section compares the method CT-cvx to its semi-convex variant CT-scvx and to other methods. At first, we consider the learning of atoms from incomplete data and image inpainting in Fig. 9. It can be seen there that for the *Patches* image, the semi-convex variant achieves an almost perfect results: It is able to learn exactly the three atoms that compose the texture part of the image and to inpaint the image very well. For the *Barbara* image, where more atoms are necessary to synthesize the texture part, the two methods yield similar results and also the atoms are similar. These results are also reflected in the PSNR values of Table 1, where CT-scvx is more that 7 decibel better for the *Patches* image and achieves only a slight improvement for *Barbara*.

**Fig. 10:**
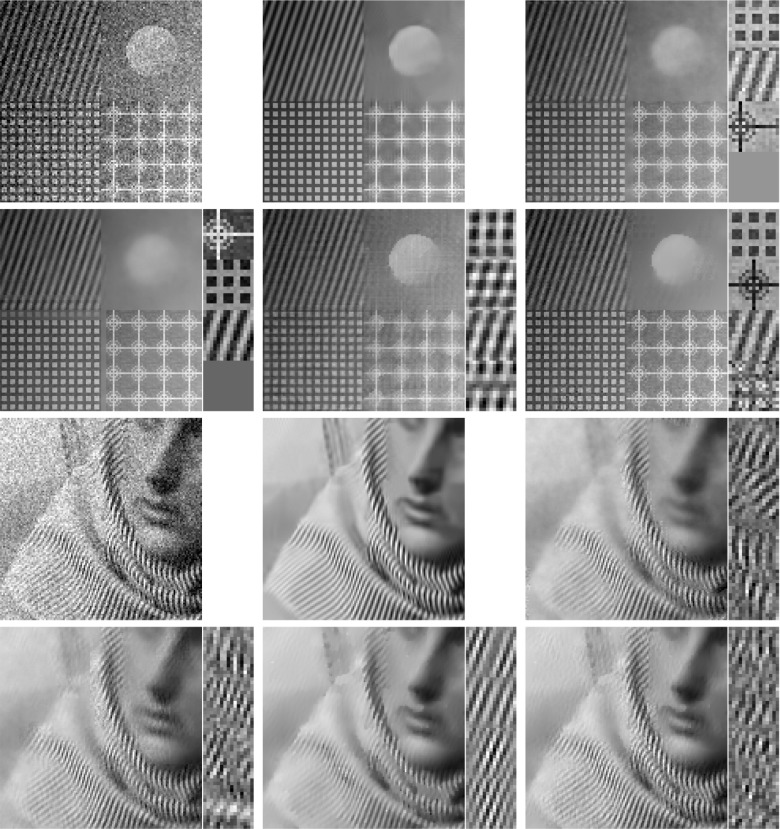
Comparison of different methods for denoising the *Patches* and *Barbara* images from Fig. 7. First row for each image, from left to right: Noisy data, BM3D, CDL. Second row for each image, from left to right: CL, CT-cvx and CT-scvx. The four most important learned atoms are shown right to the image, if applicable

Next we consider the semi-convex variant CT-scvx for denoising the *Patches* and *Barbara* images of Fig. 7. In this setting, also other methods are applicable and we compare to an own implementation of a variant of the convolutional Lasso algorithm (called CL), to BM3D denoising [22] (called BM3D) and to a reference implementation for convolutional dictionary learning (called CDL). For CL, we strive to solve the non-convex optimization problem

$$\begin{aligned}&\min _{ u, (c_i)_i, (p_i)_i } {{\,\mathrm{TV}\,}}_\rho \left( u- \sum _{i=1}^k c_i * p_i \right) + \sum _{i=1}^k \Vert c_i\Vert _1 + \left\| u - f_0 \right\| _2 ^2 \\&\quad \text {s.t. } \Vert p_i\Vert _2 \le 1, \int p_i = 0 \text { for } i=1,\ldots ,k \end{aligned}$$

where $(c_i)_i$ are coefficient images, $p_i$ are atoms and *k* is the number of used atoms. Note that we use the same boundary extension, atom-domain size and stride variable than in the methods CT-cvx, CT-scvx, and that ${{\,\mathrm{TV}\,}}_\rho$ denotes a discrete TV functional with a slight smoothing of the $L^1$ norm to make it differentiable (see the source code [16] for details). For the solution, we use an adaption of the algorithm of [48]. For BM3D, we use the implementation obtained from [34]. For CDL, we use a convolutional dictionary learning implementation provided by the SPORCO library [56, 64], and more precisely, we adapted the convolutional dictionary learning example (*cbpdndl_cns_gry*) which uses a dictionary learning algorithm (*dictlrn.cbpdndl.ConvBPDNDictLearn*) based on the ADMM consensus dictionary update [28, 57]. Note that CDL addresses the same problem as CL, however, instead of including the TV component the image is high-pass-filtered prior to dictionary learning using Tikhonov regularization.

#### Remark 16

We note that, while we provide the comparison to BM3D in order to have a reference on achievable denoising quality, we do not aim to propose an improved denoising method that is comparable to BM3D. In contrast to BM3D, our method constitutes a variational (convex) approach, that is generally applicable for inverse problems and for which we were able to provide a detailed analysis in function space such that in particular stability and convergence results for vanishing noise can be proven. Furthermore, beyond mere image reconstruction, we regard the ability of simultaneous image-atom-learning and cartoon–texture decomposition as an important feature of our approach.

Results for the *Patches* and *Barbara* image can be found in Fig. 10, where for CL and CDL we allowed for three atoms for the *Patches* images an tested 3, 5, and 7 atoms for the *Barbara* image, showing the best result that was obtained with 7 atoms. Note that for all methods, parameters were chosen and optimized according to Table 2 and for the CDL method we also tested the standard setting of 64 atoms of size $8\times 8$, which performed worse than the choice of 7 atoms. In this context, it is important to note that the implementation of CDL was designed for dictionary learning from a set of clean training images, for which it makes sense to learn a large number of atoms. When “misusing” the method for joint learning and denoising, it is natural that the number of admissible atoms needs to be constraint to achieve a regularizing effect.

**Table 2:** Parameter choice for all methods and experiments used in the paper. Here, $\lambda$ always defines the trade-off between data fidelity and regularization, $\mu$ defined the trade-off between cartoon and texture, $\nu$ defined the trade-off between the 1/2 norm and the penalization of singular values and $\epsilon$ defines the degree of non-convexity for the semi-convex potential. Whenever a parameter was optimized over a certain range for each experiment, we write *opt*

	CT-cvx			CT-scvx				TGV	TXT		BM3D	CL		CDL
	$\lambda$	$\mu$	$\nu$	$\lambda$	$\mu$	$\nu$	$\epsilon$	$\lambda$	$\lambda$	$\nu$	$\lambda$	$\lambda$	$\mu$	$\lambda$
Dcp.	–	opt	0.95						–	0.75				
Inp.	–	opt	0.975	-	opt	0.975	0.1	–	–	0.975				
Den.	opt	opt	0.975	opt	opt	0.975	2.0	opt	opt	0.975	opt	opt	opt	opt
Dcv.	opt	opt	0.975					opt						

Looking at Fig. 10, it can be seen that, as with the inpainting results, CT-scvx achieves a very strong improvement compared to CT-cvx for the *Patches* image (obtaining the atoms almost perfectly) and only a slight improvement for the *Barbara* image. Regarding the *Patches* image, the CL and CDL methods perform similar but slightly worse than CT-scvx. While there, also the three main features are identified correctly, they are not centered which leads to artifacts in the reconstruction and might be explained by the methods being stuck in a local minimum. For this image, the result of BM3D is comparable but slightly smoother than the ones of CT-scvx. In particular, the target-like structure in the bottom left is not very well reconstructed with BM3D but suffers from less remaining noise. For the *Barbara* image, BM3D delivers the best result, but a slight over-smoothing is visible. Regarding the PSNR values of Table 1, BM3D performs best and CT-scvx second best (better that CL and CDL), where in accordance with the visual results the difference of BM3D and CT-scvx for the Patches image is not as high as with *Barbara*.

## Discussion

Using lifting techniques, we have introduced a (potentially convex) variational approach for learning image atoms from corrupted and/or incomplete data. An important part of our work is the analysis of the proposed model, which shows well-posedness results in function space for a general inverse problem setting. The numerical part shows that indeed our model can effectively learn image atoms from different types of data. While this works well also in a convex setting, moving to a semi-convex setting (which is also captured by our theory) yields a further, significant improvement. While the proposed method can also be regarded solely as image reconstruction method, we believe its main feature is in fact the ability to learn image atoms from incomplete data in a mathematically well-understood framework. In this context, it is important to note that we expect our approach to work well whenever the non-cartoon part of the underlying image is well described with only a few filters. This is natural, since we learn only from a single dataset and allowing for a large number of different atoms will remove the regularization effect of our approach.

As discussed in introduction, the learning of convolutional image atoms is strongly related to a deep neural networks, in particular also when using a multilevel setting. Motivated by this, future research questions are an extension of our method in this direction as well as is exploration for classification problems.

## A Appendix: Tensor Spaces

We recall here some basic results on tensor products of Banach spaces that will be relevant for our work. Most of these results are obtained from [24, 53], to which we refer to for further information and a more complete introduction to the topic.

Throughout this section, let always $(X,\Vert \cdot \Vert _X),(Y,\Vert \cdot \Vert _Y), (Z,\Vert \cdot \Vert _Z)$ be Banach spaces. By $X^*$, we denote the analytic dual of *X*, i.e., the space of bounded linear functionals from *X* to $\mathbb {R}$. By $\mathcal {L}(X,Y)$ and $\mathcal {B} (X\times Y,Z)$, we denote the spaces of bounded linear and bilinear mappings, respectively, where the norm for the latter is given by $\Vert B\Vert _ \mathcal {B} = \sup \{ \Vert B(x,y)\Vert _Z \, \left| \right. \, \Vert x\Vert _X \le 1,\, \Vert y\Vert _Y \le 1 \}$. In case the image space is the reals, we write $\mathcal {L}(X)$ and $\mathcal {B} (X\times Y)$.

*Algebraic Tensor Product* The tensor product $x \otimes y$ of two elements $x\in X$, $y \in Y$ can be defined as a linear mapping on the space of bilinear forms on $X \times Y$ via

$$\begin{aligned} x \otimes y (A) = A(x,y), \end{aligned}$$

The algebraic tensor product $X \otimes Y$ is then defined as the subspace of the space of linear functionals on *B*(*X*, *Y*) spanned by elements $x \otimes y$ with $x\in X$, $y \in Y$.

*Tensor Norms* We will use two different tensor norms, the *projective* and the *injective* tensor norm (also known as the largest and smallest reasonable cross norm, respectively). The projective tensor norm on $X \otimes Y$ is defined for $C \in X \otimes Y$ as

$$\begin{aligned} \Vert C\Vert _\pi := \inf \left\{ \sum _{i=1}^n \Vert x_i\Vert _X \Vert y_i\Vert _Y \, \left| \right. \, C = \sum _{i=1}^n x_i \otimes y_i , \, n \in \mathbb {N}\right\} . \end{aligned}$$

Note that indeed $\Vert \cdot \Vert _\pi$ is a norm and $\Vert x \otimes y\Vert _\pi = \Vert x\Vert _X \Vert y\Vert _Y$ (see [53, Proposition 2.1]). We denote by $X \otimes _\pi Y$ the completion of the space $X \otimes Y$ equipped with this norm. The following result gives a useful representation of elements in $X \otimes _\pi Y$ and their projective norm.

### Proposition 17

For $C \in X \otimes _\pi Y$ and $\epsilon > 0$, there exist bounded sequences $(x_n)_n \subset X$, $(y_n)_n \subset Y$ such that

$$\begin{aligned} C = \sum _{i=1}^\infty x_n \otimes y_n \quad \text {and} \quad \sum _{i=1}^\infty \Vert x_n\Vert _X\Vert y_n\Vert _Y < \Vert C\Vert _\pi + \epsilon . \end{aligned}$$

In particular,

$$\begin{aligned} \Vert C\Vert _\pi = \inf \left\{ \sum _{i=1}^\infty \Vert x_i\Vert _X\Vert y_i\Vert _Y \, \left| \right. \, C = \sum _{i=1}^\infty x_i \otimes y_i \right\} . \end{aligned}$$

Now for the injective tensor norm, we note that elements of the tensor product $X \otimes Y$ can be viewed as bounded bilinear forms on $X^* \times Y^*$ by associating with a tensor $C= \sum _{i=1}^n x_i \otimes y_i$ the bilinear form $B_C(\phi ,\psi )= \sum _{i=1}^n \phi (x_i)\psi (y_i)$, where this association is unique (see [53, Section 1.3]). Hence, $X \otimes Y$ can be regarded as a subspace of $\mathcal {B} (X^* \times Y^*)$ and the injective tensor norm is the norm induced by this space. Thus, for $C= \sum _{i=1}^n x_i \otimes y_i$ the injective tensor norm $\Vert \cdot \Vert _\mathfrak {i}$ is given as

$$\begin{aligned} \Vert C\Vert _\mathfrak {i}= \sup \left\{ \left| \sum _{i=1}^n \phi (x_i)\psi (y_i) \right| \, \left| \right. \, \Vert \phi \Vert _{X^*} \le 1, \, \Vert \psi \Vert _{Y^*} \le 1\right\} \end{aligned}$$

and the injective tensor product $X \otimes _\mathfrak {i}Y$ is defined as the completion of $X \otimes Y$ with respect to this norm.

*Tensor Lifting* The next result (see [53, Theorem 2.9]) shows that there is a one-to-one correspondence between bounded bilinear mappings from $X \times Y$ to *Z* and bounded linear mappings from $X \otimes _\pi Y$ to *Z*.

### Proposition 18

For $B \in \mathcal {B} (X \times Y ,Z)$ there exists a unique linear mapping ${\hat{B}}:X \otimes _\pi Y \rightarrow Z$ such that ${\hat{B}}(x \otimes y) = B(x,y)$. Further, ${\hat{B}}$ is bounded and the mapping $B \mapsto {\hat{B}}$ is an isometric isomorphism between the Banach spaces $\mathcal {B} (X\times Y,Z)$ and $\mathcal {L}(X \otimes _\pi Y,Z)$.

Using this isometry, for $B \in \mathcal {B} (X\times Y,Z)$ we will always denote by ${\hat{B}}$ the corresponding linear mapping on the tensor product.

The following result is provided in [53, Proposition 2.3] and deals with the extension of linear operators to the tensor product.

### Proposition 19

Let $S \in \mathcal {L}(X,W)$, $T \in \mathcal {L}(Y,Z)$. Then there exists a unique operator $S \otimes _\pi T: X \otimes _\pi Y \rightarrow W \otimes _\pi Z$ such that $S \otimes _\pi T(x \otimes y) = (Sx) \otimes (Ty)$. Furthermore, $\Vert S \otimes _\pi T \Vert = \Vert S\Vert \Vert T\Vert$.

*Tensor Space Isometries* The following proposition deals with duality of the injective and the projective tensor products. To this aim, we need the notion of *Radon Nikodým property* and *approximation property*, which we will not define here but rather refer to [53, Sections 4 and 5] and [24]. For our purposes, it is important to note that both properties hold for $L^r$-spaces with $r \in (1,\infty )$, the *Radon Nikodým property* holds for reflexive spaces, but while we cannot expect the *Radon Nikodým property* to hold for $L^\infty$ and $\mathcal {M}$, the approximation property does.

### Lemma 20

Assume that either $X^*$ or $Y^*$ has the Radon Nikodým property and that either $X^*$ or $Y^*$ has the approximation property. Then

$$\begin{aligned} (X \otimes _\mathfrak {i}Y)^* \hat{=} X^* \otimes _\pi Y^* \end{aligned}$$

and for simple tensors $C = \sum _{i=1}^n x_i \otimes y_i \in X \otimes _\mathfrak {i}Y$ and $C^* = \sum _{i=1}^m x^*_i \otimes y^*_i \in X^* \otimes _\pi Y^*$ the duality pairing is given as

$$\begin{aligned} \langle C^*,C\rangle = \sum _{i=1}^n \sum _{j=1}^m \langle x_j^*,x_i \rangle \langle y_j^*,y_i \rangle \end{aligned}$$

### Proof

The identification of the duals is shown in [53, Theorem 5.33]. For the duality paring, we first note that the action of an element $C^* \in X^* \otimes _\pi Y^*$ on $X \otimes _\mathfrak {i}Y$ is given as the action of the associated bilinear form $B_{C^*}$ [53, Section 3.4], which for simple tensors $C= \sum _{i=1}^n x_i \otimes y_i$ can be given as

$$\begin{aligned} \langle B_{C^*},C\rangle = \sum _{i=1}^n B_{C^*} (x_i,y_i) . \end{aligned}$$

Now in case also $C^*$ is a simple tensor, i.e., $C^* = \sum _{i=1}^m x_i^* \otimes y_i^*$, the action of this bilinear form can be given more explicitly [53, Section 1.3], which yields

$$\begin{aligned} \langle B_{C^*},C\rangle = \sum _{i=1}^n \sum _{i=1}^m \langle x^*_i,x_i\rangle \langle y^*_i,y_i\rangle . \end{aligned}$$

$$\square $$

The duality between the injective and projective tensor products will be used for compactness assertions on subsets of the latter. To this aim, we note in the following lemma that separability of the individual space transfers to the tensor product. As a consequence, in case $X^*$ and $Y^ *$ satisfy the assumption of Lemma 20 and both admit a separable predual, also $X^ * \otimes _\pi Y^ *$ admits a separable predual and hence bounded sets are weakly* compact.

### Lemma 21

Let *X*, *Y* be separable spaces. Then both $X \otimes _\mathfrak {i}Y$ and $X \otimes _\pi Y$ are separable.

### Proof

Take $X'$ and $Y'$ to be dense countable subsets of *X* and *Y*, respectively. First note that it suffices to show that any simple tensor $x \otimes y$ can be approximated arbitrarily close by $x'\otimes y'$ with $x' \in X'$, $y' \in Y'$. But this is true since (using [53, Propositions 2.1 and 3.1])

$$\begin{aligned} \Vert x \otimes y - x'\otimes y'\Vert\le & {} \Vert x \otimes y - x\otimes y'\Vert + \Vert x \otimes y' - x'\otimes y'\Vert \\= & {} \Vert x\Vert \Vert y-y'\Vert + \Vert y'\Vert \Vert x-x'\Vert , \end{aligned}$$

where $\Vert \cdot \Vert$ denotes either the projective or the injective norm.

The following result, which can be obtained by direct modification of the result shown at the beginning of [53, Section 3.2], provides an equivalent representation of the injective tensor product in a particular case.

### Lemma 22

Denote by $C_c(\Omega _\Sigma ,X)$ the space of compactly supported continuous functions mapping from $\Omega _\Sigma$ to *X* and denote by $C_0(\Omega _\Sigma ,X)$ its completion with respect to the norm $\Vert \phi \Vert _\infty := \sup _{t \in \Omega _\Sigma } \Vert X\Vert _X$. Then, we have that

$$\begin{aligned} C_0(\Omega _\Sigma ) \otimes _\mathfrak {i}X \hat{=} C_0(\Omega _\Sigma ,X) \end{aligned}$$

where the isometry is given as the completion of the isometric mapping $J: C_0(\Omega _\Sigma ) \otimes X \rightarrow C_0(\Omega _\Sigma ,X)$ defined for $C = \sum _{i=1}^n f_i \otimes x_i$ as

$$\begin{aligned} JC(t):= \sum _{i=1}^n f_i(t)x_i . \end{aligned}$$

Next we consider the identification of tensor products with linear operators which is provided in the following proposition [53, Corollary 4.8].

### Proposition 23

Define the mapping $J:X^* \otimes _\pi Y \rightarrow \mathcal {L}(X,Y)$ as

$$\begin{aligned} C= \sum _{i=1}^\infty \phi _n \otimes y_n \mapsto L_C:X \rightarrow Y \text { where } L_C(x)= \sum _{i=1}^\infty \phi _n(x) y_n. \end{aligned}$$

Then, *J* is well-defined and has unit norm. Defining $\mathcal {N}(X,Y)\subset \mathcal {L}(X,Y)$ as the range of *J*, equipped with the norm

$$\begin{aligned} \Vert T\Vert _\text {nuc}= \inf \bigg \{ \sum _{i=1}^\infty \Vert \phi _n\Vert _{X^*}\Vert y_n\Vert _Y \, \left| \right. \, T(x) = \sum _{i=1}^\infty \phi _n(x)y_n \bigg \}, \end{aligned}$$

we get that $\mathcal {N}(X,Y)$ is a Banach space, called the space of nuclear operators. If further either $X^*$ or *Y* has the approximation property, then *J* is an isometric isomorphism, that is, we can identify

$$\begin{aligned} X^* \otimes _\pi Y = \mathcal {N}(X,Y) \end{aligned}$$

It is easy to see that nuclear operators are compact and that we can equivalently write

$$\begin{aligned}&\Vert T\Vert _\text {nuc}= \inf \bigg \{ \sum _{i=1}^\infty \sigma _i \, \left| \right. \, T(x) \\&\quad = \sum _{i=1}^\infty \sigma _i \phi _i(x)y_i, \, \Vert \phi _i\Vert _{X^*}\le 1,\, \Vert y_i\Vert _Y\le 1 \bigg \}. \end{aligned}$$

Also, in a Hilbert space setting (see [63] for details), it is a classical result that for any compact $T \in \mathcal {L}(H_1,H_2)$ with $(H_1,(\cdot ,\cdot )),(H_2(\cdot ,\cdot ))$ Hilbert spaces there exist orthonormal systems $(x_i)_i$, $(y_i)_i$ and uniquely defined singular values $(\sigma _i)_i := (\sigma _i(T))_i$ such that

$$\begin{aligned} Tx = \sum _{i=1}^\infty \sigma _i (x,x_i)y_i . \end{aligned}$$

In addition, in case *T* has finite nuclear norm, it follows that $\Vert T\Vert _\text {nuc}= \sum _{i=1}^\infty \sigma _i .$

## References

[1] Adler J, Öktem O (2018). Learned primal-dual reconstruction. IEEE Trans. Med. Imaging.

[2] Aharon M, Elad M, Bruckstein A (2006). K-SVD: An algorithm for designing overcomplete dictionaries for sparse representation. IEEE Trans. Signal Process..

[3] Ahmed A, Recht B, Romberg J (2013). Blind deconvolution using convex programming. IEEE Trans. Inf. Theory.

[4] Arora, S., Ge, R., Ma, T., Moitra, A.: Simple, efficient, and neural algorithms for sparse coding. In: Grünwald, P., Hazan, E., Kale, S. (eds.) Proceedings of The 28th Conference on Learning Theory, Proceedings of Machine Learning Research, vol. 40, pp. 113–149. PMLR (2015)

[5] Aubert G, Kornprobst P (2006). Mathematical Problems in Image Processing: Partial Differential Equations and the Calculus of Variations.

[6] Bach, F., Mairal, J., Ponce, J.: Convex sparse matrix factorizations. arXiv preprint arXiv:0812.1869 (2008)

[7] Bredies K, Holler M (2014). Regularization of linear inverse problems with total generalized variation. J. Inverse Ill Posed Probl..

[8] Bredies K, Holler M (2015). A TGV-based framework for variational image decompression, zooming and reconstruction. Part II: Numerics. SIAM J. Imaging Sci..

[9] Bredies K, Kunisch K, Pock T (2010). Total generalized variation. SIAM J. Imaging Sci..

[10] Bredies K, Lorenz DA (2009). Regularization with non-convex separable constraints. Inverse Probl..

[11] Bredies, K., Valkonen, T.: Inverse problems with second-order total generalized variation constraints. In: Proceedings of SampTA 2011—9th International Conference on Sampling Theory and Applications, Singapore (2011)

[12] Brezis H (2010). Functional Analysis, Sobolev Spaces and Partial Differential Equations.

[13] Buades A, Coll B, Morel J-M (2005). A non-local algorithm for image denoising. Proc. CVPR.

[14] Calatroni, L., Cao, C., De Los Reyes, J.C., Schönlieb, C.-B., Valkonen, T.: Bilevel approaches for learning of variational imaging models. In: Variational Methods in Imaging and Geometric Control, Radon Series on Computational and Applied Mathematics, vol. 18, pp. 252–290 (2016)

[15] Campisi P, Egiazarian K (2016). Blind Image Deconvolution: Theory and Applications.

[16] Chambolle, A., Holler, M., Pock, T.: Source code to reproduce the results of “A convex variational model for learning convolutional image atoms from incomplete data”. https://github.com/hollerm/convex_learning 10.1007/s10851-019-00919-7 PMC7138786 32300265

[17] Chambolle A, Pock T (2011). A first-order primal-dual algorithm for convex problems with applications to imaging. J. Math. Imaging Vis..

[18] Chambolle A, Pock T (2016). An introduction to continuous optimization for imaging. Acta Numer..

[19] Chandrasekaran V, Recht B, Parrilo PA, Willsky AS (2012). The convex geometry of linear inverse problems. Found. Comput. Math..

[20] Chaudhuri S, Velmurugan R, Rameshan R (2016). Blind Image Deconvolution.

[21] Chi Y (2016). Guaranteed blind sparse spikes deconvolution via lifting and convex optimization. IEEE J. Sel. Top. Signal Process..

[22] Dabov K, Foi A, Katkovnik V, Egiazarian K (2007). Image denoising by sparse 3-D transform-domain collaborative filtering. IEEE Trans. Image Process..

[23] Delon, J., Desolneux, A., Sutour, C., Viano, A.: RNLp : Mixing non-local and TV-Lp methods to remove impulse noise from images. 2017. MAP5 2016-29, Hal-preprint Nr. hal01381063v2

[24] Diestel J, Uhl JJ (1977). Vector Measures.

[25] Fazel, M.: Matrix rank minimization with applications. Ph.D. thesis, Standford University (2002)

[26] Figueiredo, M.A.: Synthesis versus analysis in patch-based image priors. In: 2017 IEEE International Conference on Acoustics, Speech and Signal Processing (ICASSP), pp. 1338–1342. IEEE (2017)

[27] Gao Y, Bredies K (2018). Infimal convolution of oscillation total generalized variation for the recovery of images with structured texture. SIAM J. Imaging Sci..

[28] Garcia-Cardona C, Wohlberg B (2018). Convolutional dictionary learning: A comparative review and new algorithms. IEEE Trans. Comput. Imaging.

[29] Haeffele, B.D., Vidal, R.: Structured low-rank matrix factorization: Global optimality, algorithms, and applications. IEEE Trans. Pattern. Anal. Mach. Intell. (2019) 10.1109/TPAMI.2019.2900306 30794507

[30] Hintermüller M, Rautenberg CN (2017). Optimal selection of the regularization function in a weighted total variation model. Part I: Modelling and theory. J. Math. Imaging Vis..

[31] Hofmann B, Kaltenbacher B, Pöschl C, Scherzer O (2007). A convergence rates result for Tikhonov regularization in Banach spaces with non-smooth operators. Inverse Probl..

[32] Holler M, Huber R, Knoll F (2018). Coupled regularization with multiple data discrepancies. Inverse Probl..

[33] Holler M, Kunisch K (2014). On infimal convolution of TV-type functionals and applications to video and image reconstruction. SIAM J. Imaging Sci..

[34] Implementation of BM3D denoising, v2.00 (30 January 2014). Obtained from http://www.cs.tut.fi/~foi/GCF-BM3D 1 Oct 2018

[35] Kobler, E., Klatzer, T., Hammernik, K., Pock, T.: Variational networks: Connecting variational methods and deep learning. In: German Conference on Pattern Recognition, pp. 281–293. Springer (2017)

[36] Kunisch K, Pock T (2013). A bilevel optimization approach for parameter learning in variational models. SIAM J. Imaging Sci..

[37] Lebrun M, Colom M, Buades A, Morel J-M (2012). Secrets of image denoising cuisine. Acta Numer..

[38] LeCun Y, Bengio Y, Hinton G (2015). Deep learning. Nature.

[39] Lewicki, M.S., Sejnowski, T.J.: Coding time-varying signals using sparse, shift-invariant representations. In: Advances in Neural Information Processing Systems, pp. 730–736 (1999)

[40] Ling S, Strohmer T (2018). Regularized gradient descent: A non-convex recipe for fast joint blind deconvolution and demixing. Inf. Inference J. IMA.

[41] Lunz, S., Öktem, O., Schönlieb, C.-B.: Adversarial regularizers in inverse problems. arXiv preprint arXiv:1805.11572 (2018)

[42] Mallat S (2009). A Wavelet Tour of Signal Processing—The Sparse Way with Contributions from Gabriel Peyré.

[43] Meyer Y (2001). Oscillating Patterns in Image Processing and Nonlinear Evolution Equations.

[44] Oymak S, Jalali A, Fazel M, Eldar YC, Hassibi B (2015). Simultaneously structured models with application to sparse and low-rank matrices. IEEE Trans. Inf. Theory.

[45] Papadopoulo, T., Lourakis, M.I.: Estimating the Jacobian of the singular value decomposition: Theory and applications. In: European Conference on Computer Vision, pp. 554–570. Springer (2000)

[46] Papyan V, Romano Y, Elad M (2017). Convolutional neural networks analyzed via convolutional sparse coding. J. Mach. Lear. Res..

[47] Papyan V, Romano Y, Sulam J, Elad M (2018). Theoretical foundations of deep learning via sparse representations: A multilayer sparse model and its connection to convolutional neural networks. IEEE Signal Process. Mag..

[48] Pock T, Sabach S (2016). Inertial proximal alternating linearized minimization (iPALM) for nonconvex and nonsmooth problems. SIAM J. Imaging Sci..

[49] Richard E, Bach FR, Vert J-P (2013). Intersecting singularities for multi-structured estimation. ICML.

[50] Richard, E., Obozinski, G.R., Vert, J.-P.: Tight convex relaxations for sparse matrix factorization. In: Advances in Neural Information Processing Systems, pp. 3284–3292 (2014)

[51] Rudin LI, Osher S, Fatemi E (1992). Nonlinear total variation based noise removal algorithms. Physica D Nonlinear Phenom.

[52] Rudin W (2017). Fourier Analysis on Groups.

[53] Ryan RA (2013). Introduction to Tensor Products of Banach Spaces.

[54] Scherzer O, Grasmair M, Grossauer H, Haltmeier M, Lenzen F (2009). Variational Methods in Imaging.

[55] Schnass K (2018). Convergence radius and sample complexity of ITKM algorithms for dictionary learning. Appl. Comput. Harmonic Anal..

[56] SParse Optimization Research COde (SPORCO), v0.1.11 (April 15, 2019). Obtained from https://github.com/bwohlberg/sporco. 26 June 2019

[57] Šorel M, Šroubek F (2016). Fast convolutional sparse coding using matrix inversion lemma. Digital Signal Process..

[58] Sulam J, Papyan V, Romano Y, Elad M (2018). Multilayer convolutional sparse modeling: Pursuit and dictionary learning. IEEE Trans. Signal Process..

[59] Sun J, Qu Q, Wright J (2016). Complete dictionary recovery over the sphere i: Overview and the geometric picture. IEEE Trans. Inf. Theory.

[60] Thompson R (1975). Singular value inequalities for matrix sums and minors. Linear Algebra Appl..

[61] Ulyanov, D., Vedaldi, A., Lempitsky, V.: Deep image prior. In: Proceedings of the IEEE Conference on Computer Vision and Pattern Recognition, pp. 9446–9454 (2018)

[62] Weickert J (1998). Anisotropic Diffusion in Image Processing.

[63] Weidmann J (1980). Linear Operators in Hilbert Spaces.

[64] Wohlberg, B.: Sporco: A python package for standard and convolutional sparse representations. In: Proceedings of the 15th Python in Science Conference, Austin, TX, USA, pp. 1–8 (2017)

[65] Zeiler, M.D., Krishnan, D., Taylor, G.W., Fergus, R.: Deconvolutional networks. In: 2010 IEEE Conference on Computer Vision and Pattern Recognition (CVPR), pp. 2528–2535. IEEE (2010)

